# Effect of Chalcogen Interaction on the Structure of Methine‐Bridged Trichalcogenophenes

**DOI:** 10.1002/chem.202501123

**Published:** 2025-06-04

**Authors:** Rio Nishimura, Ken‐ichi Yamashita

**Affiliations:** ^1^ Department of Chemistry Graduate School of Science The University of Osaka 1‐1 Machikaneyama Toyonaka Osaka 560‐0043 Japan; ^2^ Innovative Catalysis Science Division Institute for Open and Transdisciplinary Research Initiatives (ICS‐OTRI) The University of Osaka Suita Osaka 565–0871 Japan

**Keywords:** chalcogen bonding, conjugation, diastereoselectivity, noncovalent interactions, thiophene

## Abstract

Polythienylenemethylidenes (PTMs) are promising conjugated polymers for organic electronics owing to their narrow bandgaps and extended π‐conjugation. However, their stereochemistry remains unexplored. In this study, methine‐bridged trithiophene and trifuran analogs were synthesized to investigate stereochemistry and chalcogen bonding effects. The compounds were obtained as mixtures of *ZZ*, *EZ*(*= ZE*), and *EE* geometric isomers, established through detailed NMR analyses. At thermal equilibrium, the *ZZ* isomer predominated in trithiophene (*ZZ*:(*EZ + ZE*):*EE =* 58:35:6), whereas trifuran showed a near‐statistical distribution. X‐ray crystallography revealed intramolecular S···S chalcogen bonding in trithiophene with S···S distances (≈3.04 Å) shorter than van der Waals radii and C–S···S angles of 171°. Comprehensive conformer searches and DFT calculations not only validated the higher stability of the *ZZ* isomer in trithiophene but also provided calculated isomer distributions that closely matched the experimental values. Multi‐faceted computational analysis (electron localization function (ELF), noncovalent interaction (NCI), quantum theory of atoms in molecules (QTAIM), and natural bond orbital (NBO)) confirmed the presence of these chalcogen‐centered interactions and quantified their strength through lone pair (LP)(S)→σ*(S–C) donor‐acceptor orbital interactions. Trithiophene exhibited a unique dual‐chalcogen bonding mode in the *ZZ* configuration. These findings elucidate the role of chalcogen bonding in stabilizing *ZZ*‐trithiophenes and contribute to designing PTMs with controlled stereochemistry for organic electronics applications.

## Graphical abstract

The stereochemistry of methine‐bridged trichalcogenophenes was elucidated through comprehensive structural analyses. Trithiophene showed a strong preference for the *ZZ* configuration, whereas its trifuran analog exhibited a near‐statistical distribution of geometric isomers. X‐ray crystallography and computational studies revealed that intramolecular S···S chalcogen bonding stabilizes the *ZZ* configuration.

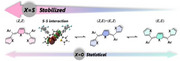

## 1 Introduction

Conjugated polymers with narrow bandgaps have attracted significant attention because of their potential applications in organic electronics,^[^ ^1^, ^2^, ^3^, ^4^, ^5^ ^]^ such as solar cells,^[^ ^6^ ^]^ field‐effect transistors,^[^ ^7^, ^8^, ^9^, ^10^ ^]^ and light‐emitting diodes.^[^ ^11^, ^12^, ^13^ ^]^ Among these materials, thiophene‐based π‐conjugated polymers represent a prominent class of conducting polymers.^[^ ^14^, ^15^, ^16^ ^]^ Polythiophenes and their derivatives have been extensively studied due to their narrow bandgap, excellent conductivity, and synthetic accessibility.

**Figure 1:**
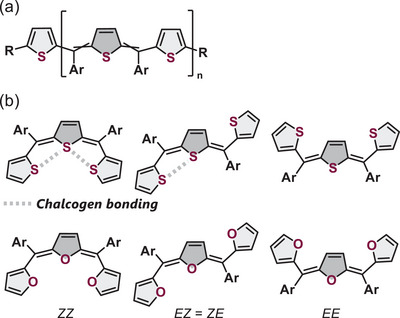
a) Structure of PTM. b) Structures of the three isomers of methine‐bridged trithiophene **1S** and trifuran **1O** investigated in this work.

Among the various thiophene‐based polymers, polythienylenemethylidenes (PTMs, Figure 1a) are a promising class of materials that feature a backbone consisting of alternating thienylene and methine units.^[^ ^17^, ^18^, ^19^, ^20^ ^]^ Their unique structure, in which oxidized quinoids alternate with thiophene units, results in a narrower bandgap compared to that of simple polythiophenes. Further bandgap reduction can be achieved by incorporating bithiophene or terthiophene units instead of a single thiophene units.^[^ ^17^, ^19^, ^20^, ^21^, ^22^ ^]^ Additionally, PTMs offer synthetic versatility through the introduction of substituents, most commonly aryl groups, at the methine carbons. This substitution capability enables the fine‐tuning of material properties. For instance, Akagi et al. demonstrated that incorporating long‐chain substituents capable of liquid crystalline behavior can enhance the solubility and stability of PTMs.^[^ ^20^, ^23^, ^24^ ^]^

Despite their promising properties, the stereochemistry of the PTMs remains largely unknown. Double bonds at the methine carbons can potentially exist in either *E*‐ or *Z*‐configurations. Previous studies on PTM stereochemistry are limited. While quantum chemical calculations on PTMs containing ethylenedioxy groups at the β‐position of thiophenes suggest a planar structure with alternating *E‐* and *Z‐* configurations, stabilized by potential S···O chalcogen bonding,^[^ ^25^ ^]^ investigations of simple PTMs are scarce. Few studies have focused on methine‐bridged trithiophene fragments^[^ ^22^, ^26^ ^]^ which can exist in three isomeric forms (*ZZ*, *EZ*/*ZE*, and *EE*) because of the *E*/*Z* isomerism of the double bonds. A well‐documented study examining trithiophenes with cyclopentadienyl anion substituents at the methine carbon demonstrated the preferential formation of the *EE* isomer through careful nuclear magnetic resonance (NMR) and nuclear Overhauser effect (NOE) analyses, attributing this preference solely to steric effects.^[^ ^27^ ^]^ Although this work provides valuable structural insights, the highly specific nature of the cyclopentadienyl anion limits its broader applicability to more conventional PTM systems. A recent report by Bonomo et al. suggested that the isomers observed in aryl‐substituted trithiophene systems were conformers arising from the rotation of the terminal thiophene rings in the *ZZ* isomer rather than geometric isomers.^[^ ^28^ ^]^

In recent years, there has been growing interest in utilizing chalcogen bonding^[^ ^29^, ^30^, ^31^ ^]^ as a powerful tool for controlling molecular conformations,^[^ ^32^, ^33^, ^34^, ^35^, ^36^, ^37^ ^]^ sensing,^[^ ^33^, ^34^, ^38^, ^39^ ^]^ and supramolecular assemblies.^[^ ^33^, ^34^, ^40^, ^41^, ^42^ ^]^ Building on these advances, in this study, we focused on the potential for chalcogen bonding between adjacent sulfur atoms in the *ZZ* configuration of methine‐bridged trithiophenes. We hypothesized that these S···S interactions might stabilize the *ZZ* isomer relative to the *EZ* and *EE* configurations. This hypothesis is supported by the well‐documented ability of thiophene sulfur atoms to participate in chalcogen bonding through their σ* orbitals along the C─S bonds, as well as their role as Lewis bases, facilitated by their LP electrons.^[^ ^43^ ^]^

To gain insights into the stereochemistry and properties of PTMs, we synthesized methine‐bridged trithiophene **1S** and its furan analog **1O** as model compounds (Figure 1b). Through detailed structural analyses, including NMR spectroscopy and X‐ray crystallography combined with theoretical calculations, we investigated the role of chalcogen bonding in determining the conformational preferences and stereochemistry of these systems. A comparison between **1S** and **1O** provides valuable insights into the chalcogen‐specific effects on the molecular structure and isomer distribution.

## 2 Results and Discussion

### 2.1 Synthesis and Characterization of the Trichalcogenophenes 1S and 1O

**Scheme 1:**
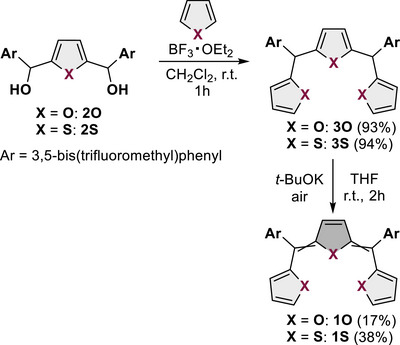
Synthesis of **1S** and **1O**.

A series of methine‐bridged trichalcogenophenes (**1X**; **X** = **S** or **O**) were synthesized via acid‐catalyzed condensation of chalcogenophenes with 2,5‐bis(hydroxymethyl) chalcogenophenes (**2X**), affording trichalcogenophene precursors (**3X**). Subsequently, these precursors were subjected to aerobic oxidation in the presence of a strong base (Scheme 1). An electron‐withdrawing aryl substituent, 3,5‐bis(trifluoromethyl)phenyl, was selected because of its ease of product synthesis and stabilization.

**Figure 2:**
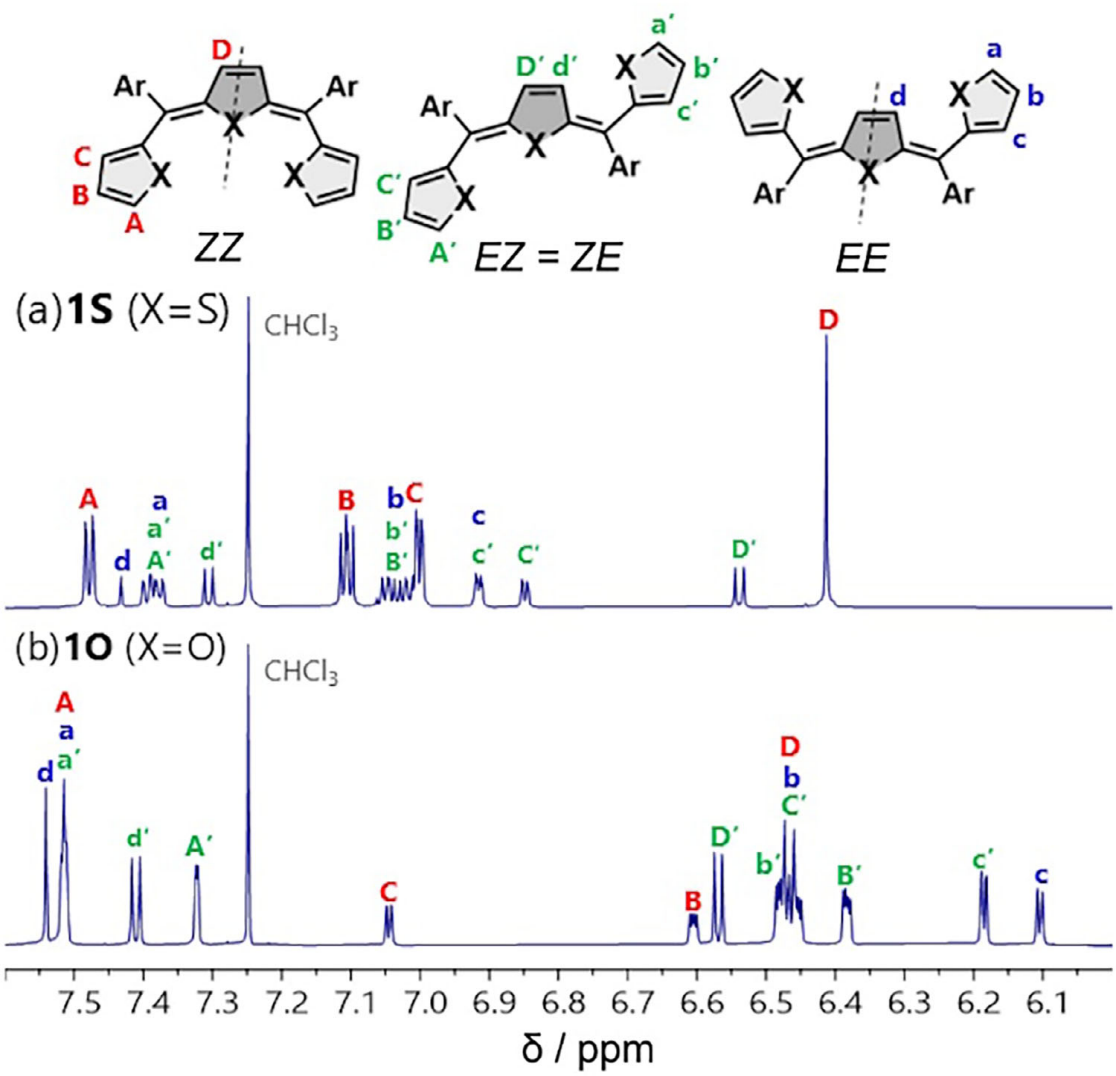
^1^H NMR (500 MHz, CDCl_3_, r.t.) spectra of geometric isomer mixtures of a) **1S** and b) **1O**.

The structures of **1X** were confirmed by NMR spectroscopy and mass spectrometry. The as‐synthesized products were mixtures of three geometric isomers, namely *ZZ*, *EZ* (= *ZE*), and *EE*, as revealed by a ^1^H NMR analysis (Figure 2). Most of the peaks observed in each spectrum were assigned using 2D ^1^H–^1^H correlation spectroscopy (COSY) and nuclear Overhauser effect spectroscopy (NOESY) measurements (Figures S8, S12, S14). The protons of the central quinoid rings showed characteristic chemical shifts depending on their proximity to the *Z*‐ or *E*‐configured double bonds. The protons adjacent to the *Z*‐configured bonds appeared upfield (e.g., 6.44 and 6.56 ppm for *ZZ*‐**1S** and *EZ*‐**1S**, respectively, in CDCl_3_) due to the shielding effect from the diamagnetic ring current of the neighboring aryl groups. In contrast, the protons near the *E*‐configured bonds resonated downfield (e.g., 7.45 and 7.33 ppm for *EE*‐**1S** and *EZ*‐**1S**, respectively, in CDCl_3_).

The ratios of the geometric isomers remained essentially unchanged at room temperature over the course of several hours (Figures S1, S2). This indicates a high activation barrier for isomerization. Specially, in the case of **1O**, when recrystallized from ethanol, *EZ*‐**1O** was selectively obtained. The ^1^H NMR spectrum of pure *EZ*‐**1O** showed negligible changes even after 3 days (Figure S2). Furthermore, in the NOESY spectra of all the compounds, no peaks originating from the chemical exchange between the isomers were observed within a mixing time of 0.5 second (Figures S8, S12, S14).

As mentioned in the Introduction, Bonomo et al. reported the presence of three isomers in similar trithiophenes based on ^1^H NMR measurements,^[^ ^38^, ^41^, ^42^ ^]^ which closely resemble our NMR data. However, they concluded that these isomers were conformers arising from the rotation of the terminal thiophene rings in the *ZZ* isomer rather than geometric isomers. The geometric nature of these isomers was established based on our 2D NMR analyses and X‐ray crystallographic studies (vide infra). Moreover, computational studies by Bonomo et al. have predicted a relatively low activation barrier of 6–7 kcal/mol for the terminal thiophene ring rotation. If this were correct, the interconversion rate would be faster than the NMR timescale, likely resulting in coalescence of the isomer signals at room temperature. Our observation of slow interconversion among the isomers also strongly supports the idea that three isomers arise from the *E/Z* configurations.

### 2.2 Ratio of Geometric Isomers

To achieve thermal equilibrium, the compounds in CDCl_3_ were equilibrated at 55 °C using the procedure described in the Supporting Information. The equilibrium ratios of the isomers were determined by ^1^H NMR spectroscopy (Figures S3, S4), and the results are summarized in Table 1. For **1O**, the ratio of *ZZ*, *EZ*, and *EE* isomers (16:64:20) approached the statistical distribution (25:50:25), suggesting minimal differences in Gibbs free energy among the three geometric isomers. In contrast, **1S** showed a marked preference for the *Z*‐configuration with a *ZZ*:*EZ*:*EE* ratio of 58:35:6.

**Table 1:** Ratio of each geometrical isomer of **1X** at equilibrium, at 55 °C.

**X**	*ZZ*	*EZ *+ *ZE*	*EE*
**O** (exp.)^[a]^	16	64	20
**O** (calcd.)^[b]^	49	39	12
**S** (exp.)^[a]^	58	35	6
**S** (calcd.)^[b]^	67	27	5

^[a]^ Determined by ^1^H NMR in CDCl_3_.

^[b]^ Determined by conformer analyses using GOAT (GFN1‐xTB) and CENSO (r^2^SCAN‐3c) program.

The geometric isomer ratios of **1S** and **1O** were also examined in solvents of varying polarity (acetone, acetonitrile, chloroform, and cyclohexane) at thermal equilibrium (55°C). As shown in Table S1, both compounds exhibited relatively consistent isomer distributions across different solvent environments. For **1S**, the *ZZ*:*EZ*:*EE* ratios showed minimal variations, supporting our hypothesis of chalcogen bonding interactions that typically remain robust in different solvent environments.^[^ ^38^, ^44^, ^45^ ^]^

We further investigated the temperature dependence of the isomer ratios to construct van't Hoff plots. By changing the solvent to [D_2_] tetrachloroethane, **1S** was equilibrated at various temperatures (60–100°C) and analyzed by ^1^H NMR as described in the Supporting Information. The results showed a gradual decrease in the *ZZ* isomer content, with a concurrent increase in the *EE* isomer content as the temperature increased (Figure S5). The thermodynamic parameters obtained via a van't Hoff analysis at five different temperatures (Figure S6) are summarized in Table S2. The Gibbs free energy differences were determined to be 0.92 ± 0.12 and 1.65 ± 0.14 kcal/mol for *ZZ*–*EZ* and *ZZ*–*EE*, respectively. Unlike **1S**, temperature‐dependent NMR analysis of **1O** could not be completed because the compound underwent thermal decomposition at the elevated temperatures.

### 2.3 Structural Analyses

We attempted to grow single crystals of **1S** for X‐ray crystallographic analyses but were unsuccessful. However, we succeeded in obtaining two single crystals from a mixture of geometric isomers of **1S′**, which is a chloro‐substituted derivative of **1S**. X‐ray crystallographic analysis revealed these to be the *ZZ* and *EZ* isomers.

The crystal of *ZZ*‐**1S′** contained two crystallographically independent molecules (molecules A and B), as shown in Figures S21, 3, respectively. The selected structural parameters are listed in Table 2. The key difference between molecules A and B lies in the conformational arrangements of the two terminal thiophenes at opposite ends of the trithiophene backbone: a *syn*‐conformation, in which the sulfur atoms of the adjacent thiophenes face each other, and an *anti*‐conformation, in which they face away. Molecule A exhibited a fixed *syn*/*anti* conformation, whereas molecule B exhibited a conformational disorder between *syn*/*syn* (86% occupancy) and *syn*/*anti* (14% occupancy). Both molecules maintained near planarity with only slight deviations. In the *syn* conformation, the S1···S2 distances were 3.0419(7) and 3.047(1) Å for molecules A and B, respectively, which are shorter than the sum of the van der Waals radii of sulfur atoms. This suggests the presence of chalcogen bonding interactions. These interactions are characteristically directional, with an ideal C–S···S angle exceeding 170°.^[^ ^43^, ^44^ ^]^ The 171° angle observed in our structures supports the presence of such interactions.

**Table 2:** Selected structural data for the crystal and DFT‐optimized structure of trithiophene **1S**.

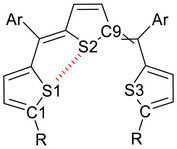				
		S1···S2 (Å)	C9–S2···S1 [°]	C1–S1···S2 [°]
X‐ray (**1S′**)	*ZZ* (A)^[a]^	3.0419(7)	174.56(7)	173.2(1)
	*ZZ* (B)^[b]^	3.047(1)	167.00(7)	170.79(9)
	*EZ*	3.0323(11)	163.47(9)	163.4(1)
DFT (**1S**)	*ZZ* ^[c]^	3.0804	167.81	164.37

^[a]^ Molecule A.

^[b]^ Molecule B.

^[c]^ Geometry optimized structure of *syn,syn‐ZZ*‐**1S** at the B3LYP‐D3BJ/def2‐TZVP level.

**Figure 3:**
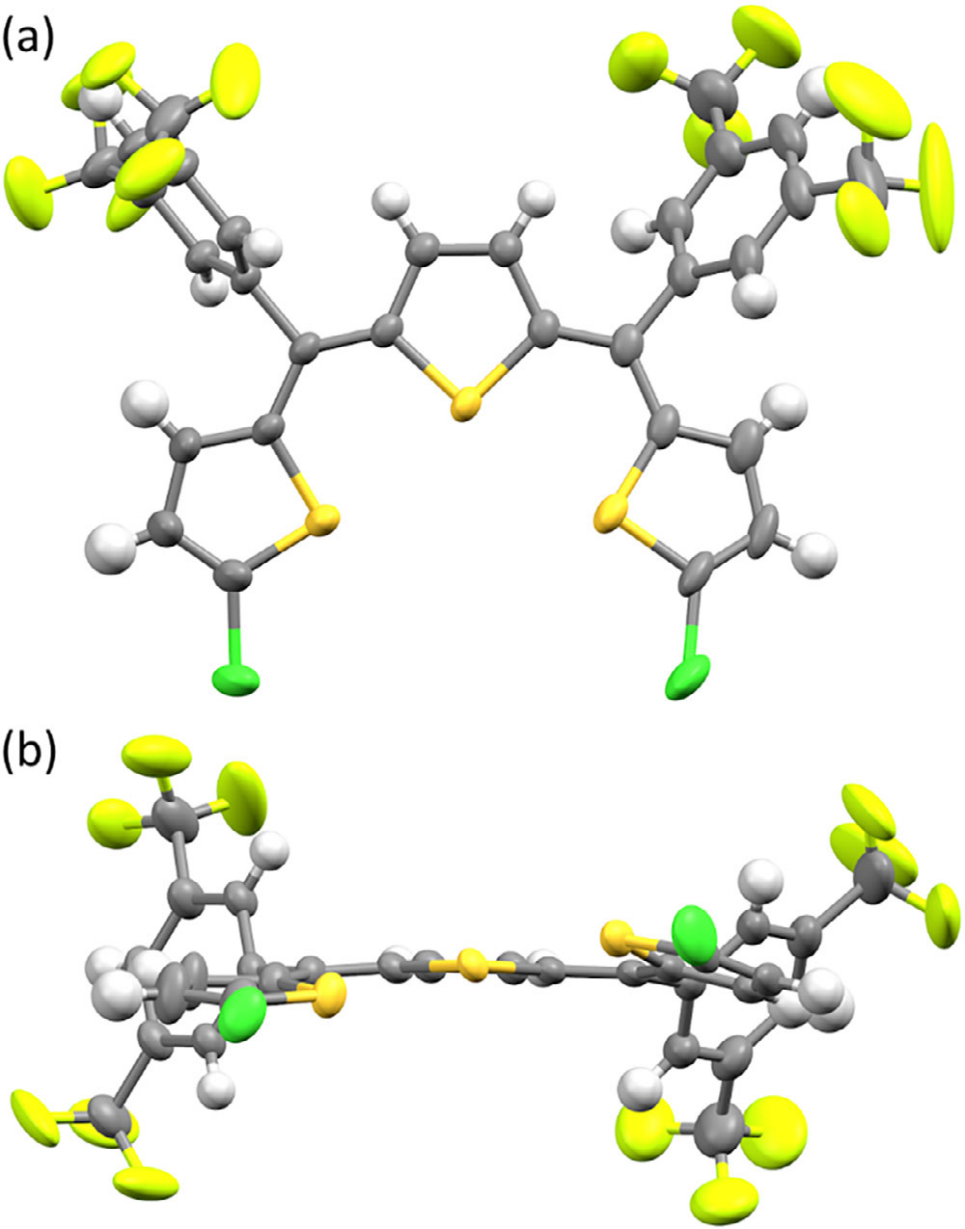
a) Top and b) side views of crystal structure of *ZZ*‐**1S′** (Molecule B) (C = gray, H = white, S = orange, F = yellow, and Cl = green). Thermal ellipsoids are drawn at 50% probability level. One of the terminal thiophene rings shows disorder between *syn‐* and *anti*‐conformations; the minor *anti*‐conformation is omitted for clarity.

The crystal structure of *EZ*‐**1S′** (Figure S22) shows that the terminal thiophene on the *Z* side also adopts a *syn* conformation, with a comparable S1···S2 distance of 3.032(1) Å.

For **1O**, the initial crystallization yielded only needle‐like crystal bundles of the *EZ* isomer (confirmed by NMR analysis), which were unsuitable for X‐ray analyses. However, subsequent crystallization of the mother liquor after the removal of the needle crystals produced single crystals that were successfully characterized by X‐ray diffraction. The crystal contained two crystallographically independent molecules, both of which were *EE* isomers with nearly identical structures (Figure S23). The structure shows three nearly coplanar furan rings.

### 2.4 Structure and Energy by Theoretical Calculations

**Figure 4:**
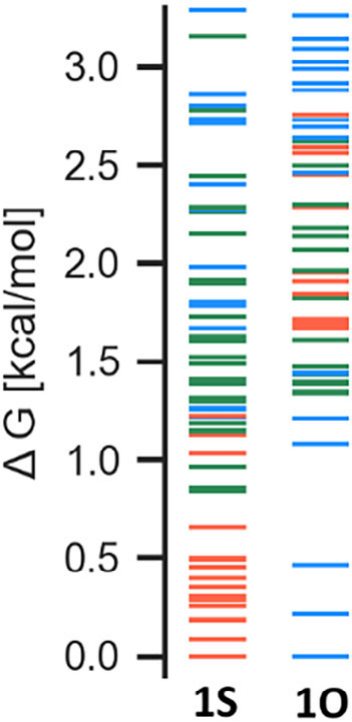
Relative Gibbs free energy (referenced to the most stable conformer) of the conformer ensembles of **1S** and **1O**, optimized using the CENSO program. Red: *ZZ*‐configuration, green: *EZ*‐configuration, and blue: *EE*‐configuration. Temperature: 55 °C.

To understand the solution‐phase *E*/*Z* isomer distributions of **1S** and **1O** and determine the most stable structures, we performed quantum chemical calculations. As per the NMR and X‐ray crystallography results, these molecules are expected to have multiple conformers for each geometric isomer owing to the rotation of the terminal thiophene and aryl substituents. This conformational complexity renders manual stable conformer searches impractical. Therefore, we developed our own modifications to the conformer search and Gibbs energy calculation protocols originally reported by Grimme et al. In the original protocol, Conformer–Rotamer Ensemble Sampling Tool (CREST)^[^ ^46^, ^47^ ^]^ was used for the initial conformer search, followed by energy refinement using the command line energetic sorting (CENSO) program.^[^ ^48^ ^]^ In our approach, we employed the global optimizer algorithm (GOAT)^[^ ^49^ ^]^ implemented in ORCA 6.0.1^[^ ^50^, ^51^ ^]^ for the initial conformer search because it does not distinguish between single and double bonds by default, allowing us to consider both conformations and configurations in a single calculation. The GOAT was executed to simultaneously explore all geometric isomer/conformer ensembles within a 12 kcal/mol threshold of Gibbs free energy (at 55 °C) at the semi‐empirical GFN1‐xTB^[^ ^52^, ^53^ ^]^ calculation level. The resulting ensemble was subsequently refined to the r^2^SCAN‐3c^[^ ^54^ ^]^ level using the CENSO program. The relative Gibbs free energies at 55 °C of the conformer ensembles of **1S** and **1O** are shown in Figure 4, while selected conformers of **1S** and **1O** are shown in Figures S24, S25, respectively.

For **1S**, all structures within 0.7 kcal/mol of the most stable structure were *ZZ* isomers. The most stable conformation of *ZZ* was the *syn*/*syn* conformation. The relative energies of the most stable *EZ* and *EE* conformers were 0.84 and 1.22 kcal/mol, respectively. These values are in reasonable agreement with those obtained from the van't Hoff analysis. Based on the calculated Gibbs free energies of the conformer ensemble, the predicted distribution ratio of *ZZ:EZ:EE* was 67:27:5, which closely matches the experimental values. This agreement validates the computational approach.

Conversely, for **1O**, the most stable structure was found of the *EE* isomer. The relative energies of the most stable *EZ* and *ZZ* conformers were 1.33 and 1.67 kcal/mol, respectively. Notably, within the *ZZ* isomer of **1O**, the *syn/syn* conformation that was most stable for **1S** was found to be less stable than the *syn/anti* and *anti/anti* conformations (Figure S25). This destabilization can be attributed to the repulsion between the LPs of adjacent oxygen atoms. The distribution ratio of *ZZ:EZ:EE*, calculated based on the Gibbs free energies of the ensemble, was 11:39:49, which also approximately matched the experimental values.

### 2.5 Theoretical Analyses of Noncovalent Interactions

**Figure 5:**
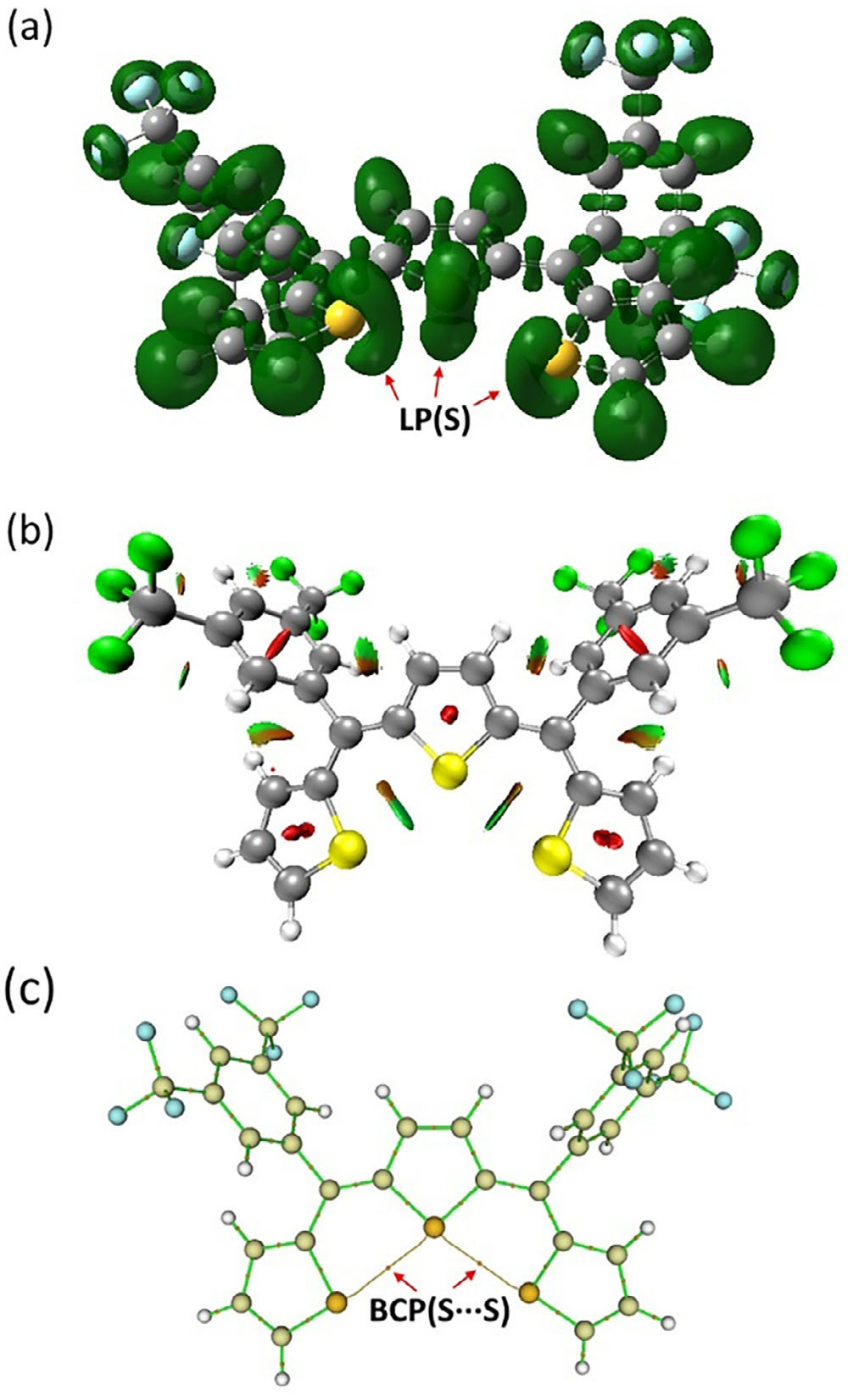
a) ELF isosurfaces (0.85 a.u.), b) NCI isosurfaces (0.5 a.u.), and c) the result of QTAIM of *syn,syn*‐*ZZ*‐**1S**.

To elucidate the nature of the interactions between adjacent chalcogen atoms in *syn,syn*‐*ZZ*‐**1S**, a multi‐faceted computational analysis approach was employed. First, ELF analysis^[^ ^55^ ^]^ was performed to visualize the distribution of electron LPs on the sulfur atoms (Figure 5a). The ELF isosurfaces (0.85 a.u.) revealed that the LPs of all three sulfur atoms display a semi‐circular distribution around each sulfur atom, similar to previously reported thiophene structures.^[^ ^43^ ^]^ As shown in Table 2, the C1‐S1···S2 angles in both crystal structures and DFT‐optimized geometry (ranging from approximately 164° to 174°) enable effective interactions.

NCI analysis^[^ ^56^ ^]^ (Figure 5b) confirmed attractive interactions between adjacent sulfur atoms, which are considered to be weak interactions, similar to van der Waals forces. These S···S interactions provide additional support for the observed conformational preferences in these systems.

The QTAIM analysis^[^ ^57^, ^58^, ^59^, ^60^ ^]^ provided additional evidence for chalcogen bonding through the identification of bond critical points (BCPs) and bond paths between sulfur atoms (Figure 5c). Table 3 summarizes the topological parameters at the S···S BCPs in *syn,syn*‐*ZZ*‐**1S**. The electron density (*ρ*) and Laplacian (∇^2^ *ρ*) values at these BCPs were 0.0153 and 0.0477 a.u., respectively. Notably, the positive value of the energy density^[^ ^60^ ^]^ (*H* = 0.0013 a.u.), the |*V*|/*G* ratio less than 1^[^ ^60^, ^61^ ^]^ (0.88), and the low eta index^[^ ^60^, ^62^ ^]^ (*η* = 0.16) are all characteristic features of closed‐shell interactions. The combination of these topological parameters definitively classifies the S···S interactions in our system as noncovalent chalcogen bonds rather than covalent or partially covalent interactions. These values fall within the expected range for weak but significant chalcogen bonding interactions that contribute substantially to the stabilization of the *ZZ* configuration.

**Table 3:** Topological parameters (in a.u.) at the S···S (BCPs) in *syn,syn*‐*ZZ*‐**1S**.

*ρ*	∇^2^ *ρ*	*H*	|*V*|/*G*	*η*
0.0153	0.0477	0.0013	0.88	0.16

*ρ*: electron density, *∇* ^2^ *ρ*: Laplacian of electron density, *H*: energy density,*V*: potential energy density, *G*: Lagrangian kinetic energy, *η*: eta index (= |*λ*_1_|/*λ*_3_, *λ*: curvature values)

**Figure 6:**
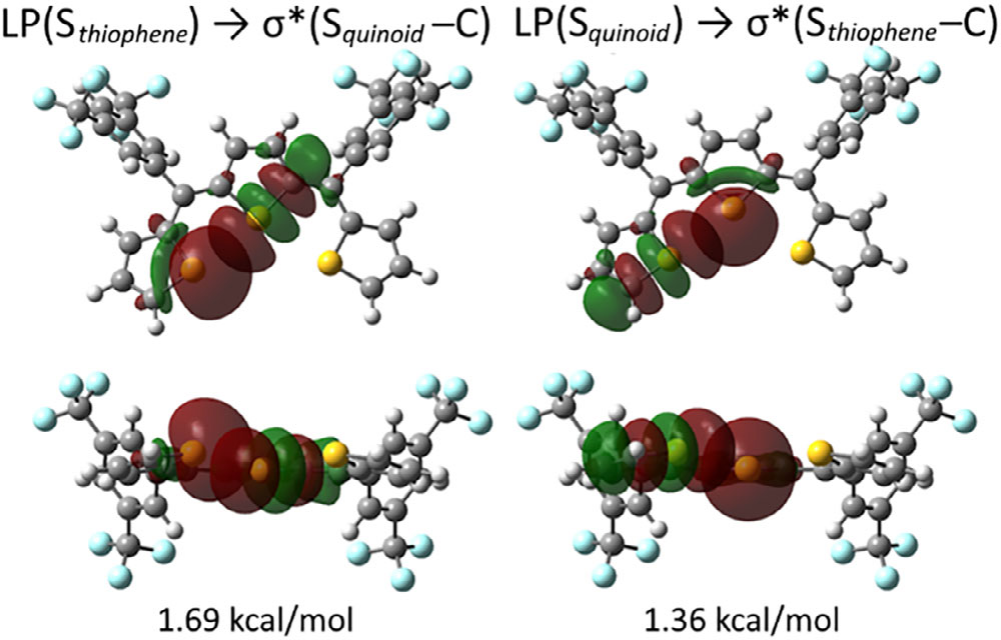
Top and side views of the calculated NBOs and second‐order perturbation energy (*E*(2), kcal/mol) involved in the chalcogen bonding in *syn,syn*‐*ZZ*‐**1S**.

The intramolecular chalcogen bonding was further investigated using NBO calculations.^[^ ^63^, ^64^ ^]^ Figure 6 shows the orbital interactions and their corresponding energies for *syn,syn*‐*ZZ‐* **1S**. Two distinct donor‐acceptor interactions were identified for each S···S pair in the *syn* conformation. In the first interaction, the LP of the sulfur atom in the terminal thiophene acted as a donor to the σ*(S–C) orbital of the sulfur in the central quinoid moiety (stabilization energy *E*(2) = 1.69 kcal/mol). The second interaction exhibited a reversed donor‐acceptor relationship with *E*(2) = 1.36 kcal/mol. This asymmetry in the stabilization energies can be attributed to the electronic nature of the respective rings, that is, electron‐rich thiophenes versus the electron‐deficient quinoid core. While the individual interaction energies are lower than those typically observed for chalcogen bonds involving thiophene units (approximately 2–8 kcal/mol),^[^ ^45^, ^65^, ^66^ ^]^ their combined effect approaches the magnitude of a conventional chalcogen bond.

Notably, *syn,syn*‐*ZZ*‐**1S** exhibits a dual‐chalcogen bonding interaction mode,^[^ ^67^, ^68^ ^]^ where the LP of the sulfur atom in the central quinoid moiety simultaneously interacts with the σ*(S–C) orbitals on both sides.

### 2.6 Comparison with Chalcogen Bonding in Simple Thiophene Homodimer Systems

The chalcogen bonding observed in our *syn,syn*‐*ZZ*‐**1S** system differs significantly from those reported in simple thiophene homodimer systems. Recent studies by Scheiner have investigated various modes of chalcogen bonding in chalcogenophene homodimers.^[^ ^43^ ^]^ However, the arrangement in our methine‐bridged trithiophene represents a distinct chalcogen bonding mode with several notable differences.

First, the S···S distances in our system (approximately 3.04 Å) are considerably shorter than those observed in Scheiner's thiophene dimers (approximately 3.5 Å across all geometries). This shorter distance indicates stronger chalcogen interactions in our methine‐bridged system. Moreover, the interaction energies reported for S···S chalcogen bonds in thiophene dimers are quite modest, with NBO *E*(2) values of only 0.31 kcal/mol in the most favorable cases. In contrast, our system exhibits significantly stronger interactions, with *E*(2) values of 1.69 and 1.36 kcal/mol for each S···S interaction.

The enhanced chalcogen bonding in our system can be primarily attributed to the methine bridge, which elegantly overcomes the steric limitations inherent in simple thiophene systems. In simple thiophene homodimers, any attempt to arrange two thiophene units in an orientation analogous to our *syn,syn*‐*ZZ*‐1S would result in severe steric hindrance between the α‐position hydrogen atoms. The methine‐bridge provides the ideal separation and angle between adjacent thiophene units, enabling optimal S···S interactions. Additionally, the electronic properties of the central quinoid structure may also contribute to the observed chalcogen bonding enhancement.

The dual donor‐acceptor role played by each sulfur atom in our system is similar to what Scheiner described as “duplex” chalcogen bonding, but achieves this within a single molecule rather than between separate molecules. This intramolecular chalcogen bonding arrangement contributes significantly to the stabilization of the *ZZ* configuration in trithiophenes and represents a unique structural feature of PTM systems.

## 3 Conclusion

In summary, we synthesized and characterized a series of methine‐bridged trichalcogenophenes, **1S** and **1O,** as model compounds to investigate the stereochemistry and chalcogen bonding effects in PTMs. The synthesized compounds were obtained as mixtures of the geometric isomers *ZZ*, *EZ*, and *EE*, whose identities were unambiguously established through detailed NMR analyses. The thermal equilibrium ratios of these isomers showed distinct patterns depending on the chalcogen atom: **1S** exhibited a strong preference for the *ZZ* isomer, whereas **1O** showed a near‐statistical distribution.

An X‐ray crystallographic analysis of a **1S′** derivative revealed the presence of intramolecular S···S chalcogen bonding interactions in both *ZZ* and *EZ* isomers. In the *ZZ* isomer, the S···S distances (approximately 3.04 Å) were shorter than the sum of the van der Waals radii, with near‐ideal C–S···S angles of 171°, strongly supporting the presence of chalcogen bonding. Comprehensive conformer searches and DFT calculations not only validated the higher stability of the *ZZ* isomer in **1S** but also provided calculated isomer distributions that closely matched the experimental values.

The presence and nature of these chalcogen bonding interactions in *syn,syn*‐*ZZ*‐**1S** were further characterized using a multi‐faceted computational analysis (ELF, NCI, QTAIM, and NBO). The NBO analysis revealed two distinct types of donor‐acceptor interactions between adjacent sulfur atoms, involving LP(S)→σ*(S–C) orbital interactions with stabilization energies of 1.69 and 1.36 kcal/mol. Significantly, this bonding arrangement represents a unique intramolecular version of the “duplex” chalcogen bonding mode, which is facilitated by the methine bridges between thiophene units. The methine‐bridge architecture enables S···S interactions that would be sterically hindered in simple thiophene homodimers, resulting in stronger chalcogen bonds with shorter S···S distances compared to those reported in thiophene dimers. Notably, *syn,syn*‐*ZZ*‐**1S** exhibited a unique dual‐chalcogen bonding interaction mode, in which the central sulfur atom simultaneously participated in the interactions on both sides.

These findings provide comprehensive experimental and theoretical evidence of the role of intramolecular chalcogen bonding in stabilizing the *ZZ* configuration in methine‐bridged trithiophenes. The insights gained from this study, particularly regarding the unique chalcogen bonding mode enabled by the methine‐bridged architecture, are expected to contribute to the understanding and design of PTMs and related oligothiophenes with controlled stereochemistry and enhanced electronic properties for organic electronic applications.

## Supporting Information

The authors have cited additional references within the Supporting Information.^[^ ^69^, ^70^, ^71^, ^72^, ^73^, ^74^, ^75^, ^76^, ^77^ ^]^ Deposition Numbers 2388514 (for *ZZ*‐**1S′**), 2388513 (for *EZ*‐**1S′**), 2388515 (for *EE*‐**1O**) contains the supplementary crystallographic data for this paper. These data are provided free of charge by the joint Cambridge Crystallographic Data Centre and Fachinformationszentrum Karlsruhe Access Structures service.

## Conflict of Interests

The authors declare no conflict of interest.

## Supporting information



Supporting Information

## Data Availability

The data that support the findings of this study are available in the supplementary material of this article.
