# Supplementary material for: Effect of Chalcogen Interaction on the Structure of Methine‐Bridged Trichalcogenophenes
Source: Chemistry. 2025 Jun 4;31(40):e202501123. doi: 10.1002/chem.202501123 (PMC12271992; doi:10.1002/chem.202501123)
Supplement: Supplementary file 1 — Supporting Information [file CHEM-31-e202501123-s001.pdf]

Contents

1. Experimental details 2
2. Additional spectroscopic data 8
3. X-Ray crystal structure determinations 27
4. Theoretical calculations 30

1. Experimental details

Instrumentation and Materials

Unless otherwise noted, all the reactions were performed in a nitrogen atmosphere using anhydrous solvents and heat-gun-dried glassware on a dual-manifold Schlenk line. Furan was distilled before use. The other chemicals were of reagent grade and were used without any further purification unless otherwise noted. Thin-layer chromatography (TLC) was performed on silica gel 60 F_{254} plates (Merck). Column chromatography was performed using silica gel 60N (Kanto Chemical, spherical, neutral, 63–210 μm). All NMR spectral data were recorded on a JEOL ECA-500 (500 MHz) or JEOL ECA-400 (400 MHz) spectrometer at ambient temperature (25 °C). ^{1}H NMR spectra were referenced internally to tetramethylsilane as a standard. ^{13}C NMR spectra were referenced internally to a solvent signal (δ = 77.0 ppm for $CDCl_3$, δ = 29.8 ppm for $(CD_3)_2CO$). High-resolution electrospray ionization mass spectrometry (ESI HRMS) data were obtained using a Thermo Fisher Scientific Q-Exactive mass spectrometer.

Synthesis of 2,5-bis((3,5-bis(trifluoromethyl)phenyl)hydroxymethyl)furan (2O)

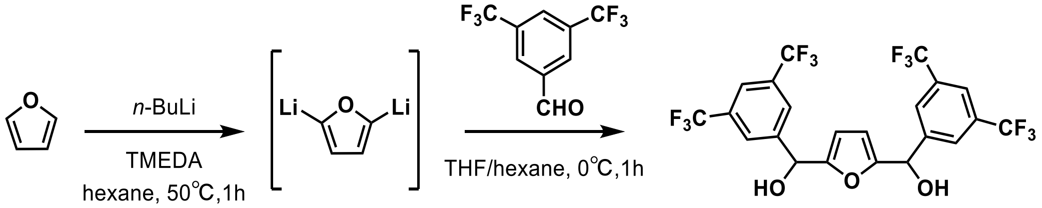

To a 100-mL two-necked flask, *n*-hexane (25 mL), *N*,*N*,*N'*,*N'*-tetramethylethylenediamine (3.8 mL, 2.5 eq.) and *n*-BuLi (2.66 M solution in hexane, 12 mL, 3.2 eq.) were added. After stirring at room temperature for 10 min, furan (0.75 mL, 1 eq.) was added to the mixture and stirred for another 60 min while heating to 50°C. After the reaction solution was cooled in an ice bath, a solution of 3,5-bis(trifluoromethyl)benzaldehyde (4.2 mL,2.5 eq.) in THF (25 mL) was added dropwise and stirred for 60 min. The solution was quenched with aqueous ammonium chloride solution. The reaction solution was diluted with ethyl acetate, washed with brine, dried over Na_2SO_4, and concentrated under reduced pressure. The crude product obtained was dissolved in methanol, then aqueous $NaHSO_3$ solution was added with stirring. The mixture was extracted with ethyl acetate. After washing with water and brine, dried over Na_2SO_4, and concentrated under reduced pressure. The product was purified by column chromatography (silica gel 60N, hexane:ethyl acetate = 4:1). Finally, the product was recrystallized from a mixture of hexane and dichloromethane to give an ochre-colored powder (1.45 g, 26%). Its spectroscopic data were consistent with previously reported ones.[69]

Synthesis of 2,5-bis((3,5-bis(trifluoromethyl)phenyl)hydroxymethyl)thiophene (2S)

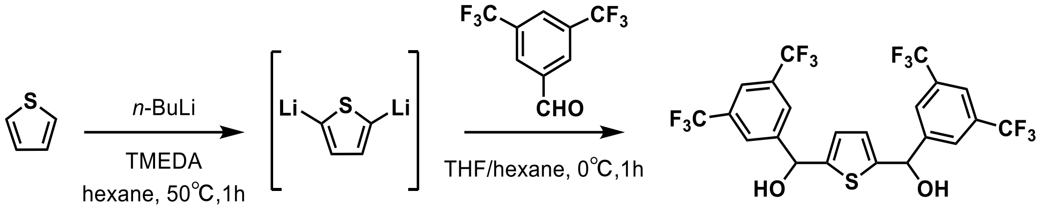

To a 100-mL two-necked flask, *n*-hexane (25 mL), *N*,*N*,*N'*,*N'*-tetramethylethylenediamine (3.8 mL, 2.5 eq.) and *n*-BuLi (2.66 M solution in hexane, 12 mL, 3.2 eq.) were added. After stirring at room temperature for 10 min, thiophene (0.80 mL, 1 eq.) was added to the mixture and stirred for another 60 min while heating to 50°C. After the reaction solution was cooled in an ice bath, a solution of 3,5-bis(trifluoromethyl)benzaldehyde (4.2 mL, 2.5 eq.) in THF (25 mL) was added dropwise and stirred for 60 min. The solution was quenched with aqueous ammonium chloride solution. The reaction solution was diluted with ethyl acetate, washed with brine, dried over Na_2SO_4. and concentrated under reduced pressure. The crude product obtained was dissolved in methanol, aqueous $NaHSO_3$ solution was added and stirred. The mixture was extracted with ethyl acetate. After washing with water and brine, dried over Na_2SO_4, and concentrated under reduced pressure. Finally, the product was recrystallized from hexane solution to give an ochre-colored powder (5.3 g, 92%). The NMR analysis suggested that the product was obtained as a mixture of diastereomers. Its spectroscopic data were consistent with previously reported ones.[70]

Synthesis of 2,5-bis((3,5-bis(trifluoromethyl)phenyl)(2-furyl)methyl)furan (3O)

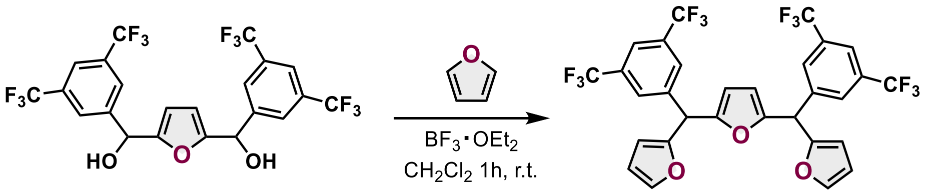

To a 200 mL two-necked flask containing **2O** (498.3 mg 0.90 mmol, 1 eq.), dichloromethane (100 mL), Furan (3.3 mL, 45 mmol, 50 eq.) and $BF_3 \cdot OEt_2$(60 µL, 0.45 mmol, 0.5 eq.) were added and stirred at room temperature for 60 min. Upon addition of $BF_3 \cdot OEt_2$, the reaction solution immediately turned brown, and then turned black-green after 60 min. After the reaction, the solution was washed with aqueous $NaHCO_3$ solution, water, and then brine. The organic phase was dried over Na_2SO_4, filtered, and concentrated under reduced pressure. The product was obtained as brown oil (551.0 mg, 93%). Its spectroscopic data were consistent with previously reported ones.[69]

Synthesis of 2,5-bis((3,5-bis(trifluoromethyl)phenyl)(2-thienyl)methyl)thiophene (3S)

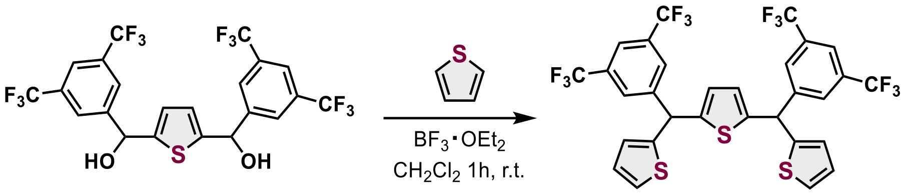

To a 200 mL two-necked flask containing **2S** (514.8 mg 0.906 mmol, 1 eq.), dichloromethane (88 mL), thiophene (1.4 mL, 18 mmol, 19 eq.) and $BF_3{\cdot}OEt_2$ (220 µL, 1.76 mmol, 1.9 eq.) were added and stirred at room temperature for 60 minutes. Upon addition of $BF_3{\cdot}OEt_2$, the reaction solution immediately turned brown, and then turned black-green after 60 min. After the reaction, the solution was washed with aqueous $NaHCO_3$ solution, water, and then brine. The organic phase was dried over Na_2SO_4, filtered, and concentrated under reduced pressure. The product was obtained as a brown oil (597.1 mg, 94%). The NMR analysis suggested that the product was obtained as a mixture of diastereomers. ^{1}H NMR (500 MHz, $CDCl_3$) δ = 7.81 (s, 2H), 7.76 (s, 4H), 7.27 – 7.20 (m, 2H), 6.99 – 6.90 (m, 2H), 6.87 – 6.78 (m, 2H), 6.71 – 6.66 (m, 2H), 5.93 (s, 2H); ^{13}C NMR (125 MHz, $CDCl_3$) δ (ppm) = 145.9 (*C*q), 144.9 (*C*q),132.1 (q, *J*= 33.4 Hz, *C*q) 128.6 (*C*H), 127.1 (*C*H), 126.9 (*C*H), 126.7 (*C*H), 126.7 (*C*H), 125.8 (*C*H), 123.4 (q, J= 266.6 Hz, *C*F$_3$), 121.6 (*C*H), 47.3 (*C*H); HRMS(ESI): *m/z* calcd for $C_{30}H_{15}F_{12}S_3$: 699.0150 ([M–H]$^-$), found 699.0155.

Synthesis of 2,5-bis((3,5-bis(trifluoromethyl)phenyl)(5-chloro-2-thienyl)methyl)thiophene (3S′)

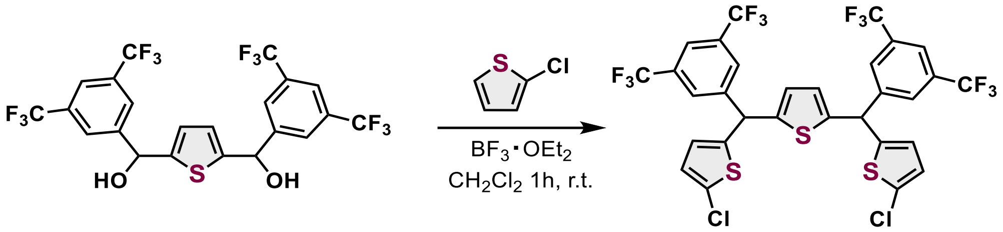

To a 100 mL two-necked flask containing **2S** (300.2 mg 0.5282 mmol, 1 eq), dichloromethane (53 mL), 2-chlorothiophene (170 µL, 1.849 mmol, 3.5 eq) and $BF_3{\cdot}OEt_2$ (135 µL, 0.5377 mmol, 1.0 eq) were added and stirred at room temperature for 60 min. Upon addition of $BF_3{\cdot}OEt_2$, the reaction solution immediately turned black-purple. After the reaction, the solution was washed with aqueous $NaHCO_3$ solution, water, and then brine. The organic phase was dried over Na_2SO_4, filtered, and concentrated under reduced pressure. The product was obtained as a brown oil (393.4 mg, 97%). The NMR analysis suggested that the product was obtained as a mixture of diastereomers. ^{1}H NMR (500 MHz, $CDCl_3$) δ = 7.81 (s, 2H), 7.70 (s, 4H), 6.82 – 6.76 (m, 2H), 6.71 – 6.65 (m, 2H), 6.59 – 6.54 (m, 2H), 5.79 (s, 2H); ^{13}C NMR (125 MHz, $CDCl_3$) δ (ppm) = 145.1 (*C*q), 145.1 (*C*q), 144.8 (*C*q), 143.3 (*C*q), 132.2 (q, *J* = 33.6 Hz, *C*q), 130.4 (*C*q), 128.4 (*C*H), 126.8 (*C*H), 126.1 (*C*H), 123.1 (q, *J* = 273.2 Hz, *C*F$_3$), 121.7 (*C*H), 47.4 (*C*H); HRMS(ESI): *m/z* calcd for $C_{30}H_{15}F_{12}S_3$: 766.93703 ([M–

H]⁻), found 766.93821.

Synthesis of 1O

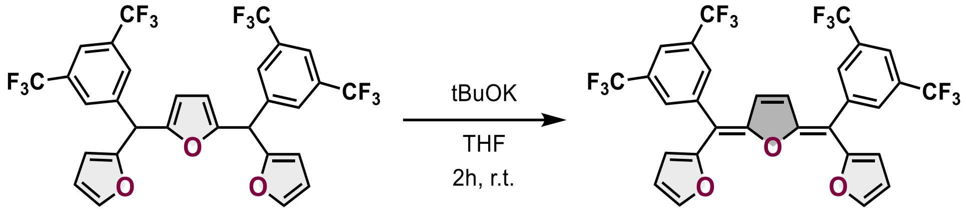

To a 300 mL flask, **3O** (551.0 mg, 0.844 mmol, 1 eq), THF (84 mL) and *t*-BuOK (236.9 mg, 2.111 mmol, 2.513 eq) were added and stirred for 120 min under room temperature at ambient condition. The reaction product was concentrated under reduced pressure, and then purified twice by column chromatography (silica gel 60N, hexane) to afford an orange solid of **1O** as a mixture of three geometric isomers (88.7 mg, 17%). Recrystallization of the products from hot ethanol yielded needle-like crystals of pure *EZ*-**1O**. The NMR data below corresponds to pure *EZ*-**1O**, while the NMR spectra of the isomer mixture are shown in Figures S11 and S12.

^{1}H NMR (500 MHz, $CDCl_3$) δ = 8.10 (s, 2H), 7.89 (s, 2H), 7.83 (s, 2H), 7.54 – 7.50 (m, 1H), 7.41 (d, J = 5.9 Hz, 1H), 7.35 – 7.31 (m, 1H), 6.57 (d, J = 5.7 Hz, 1H), 6.51 – 6.48 (m, 1H), 6.47 (d, J = 3.6 Hz, 1H), 6.41 – 6.36 (m, 1H), 6.19 (d, J = 3.4 Hz, 1H); ^{13}C NMR (125 MHz, $CDCl_3$) δ (ppm) = 155.6 (*C*q), 153.5 (*C*q), 151.7 (*C*q), 149.8 (*C*q), 143.1 (*C*H), 142.9 (*C*H), 138.3 (*C*q), 137.9 (*C*q), 131.9 (q, J = 33.4 Hz, 2Cq), 131.1 (*C*H), 130.4 (*C*H), 127.7 (*C*H), 127.5 (*C*H), 123.5 (q, J= 273.6 Hz, *C*q), 123.3 (q, J = 271.6 Hz, CF_3), 121.8 (*C*H), 121.5 (*C*H), 112.4 (*C*H), 111.9 (*C*H), 111.6 (*C*H), 111.2 (*C*H), 106.5 (*C*q), 105.7 (*C*q); HRMS(ESI): *m/z* calcd for $C_{30}H_{14}F_{12}O_3$: 650.07458 ([M]$^+$), found 650.0747; E.A.: calcd. for $C_{30}H_{14}F_{12}O_3$: C, 55.40; H, 2.17; N, 0; found: C, 55.36; H, 2.49; N, 0.

Synthesis of 1S

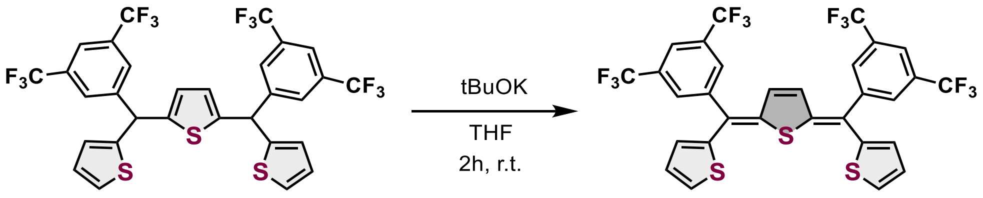

To a 200 mL flask, **3S** (560.7 mg, 0.8003 mmol, 1 eq), THF (80 mL) and *t*-BuOK (228.0 mg, 2.032 mmol, 2.539 eq) were added and stirred for 120 min under room temperature at ambient condition. The reaction product was concentrated under reduced pressure, and then purified by column chromatography (silica gel 60N, hexane) to afford an orange solid of **1S** as a mixture of three geometric isomers (114 mg, 20%). The ^{13}C NMR spectrum was further complicated by the existence of diastereomer and splitting of signals due to coupling with ^{19}F nuclei. Due to these factors, the ^{13}C NMR

data for **1S** are not provided here, and the spectrum is presented in Figure S15. ^{1}H NMR (500 MHz, $CDCl_3$) δ = 7.99 (s, Ar), 7.93 (s, Ar), 7.92 (s, Ar), 7.89 (s, Ar), 7.87 (s, Ar), 7.83 (s, Ar), 7.82 (s, Ar), 7.80 (s, Ar), 7.48 (dd, *J* = 5.1, 1.1 Hz, *ZZ*), 7.45 (s, *EE*), 7.41–7.38 (m, *EZ*), 7.32 (d, *J* = 6.2 Hz, *EZ*), 7.11 (dd, *J* = 5.0, 3.9 Hz, *ZZ*), 7.07 – 7.03 (m, *EZ*), 7.02 – 7.01 (m, *ZZ*), 6.95 – 6.91 (m, *EZ* and *EE*), 6.87 (dd, *J* = 3.8 Hz, 1.1 Hz, *EZ*), 6.56 (d, *J* = 6.1 Hz, *EZ*), 6.44 (s, *ZZ*); HRMS(ESI): *m/z* calcd for $C_{30}H_{14}F_{12}S_3$: 698.0061 ([M]$^+$), found 698.0062; E.A.: calcd. for $C_{30}H_{14}F_{12}S_3$: C, 51.58; H, 2.02; N, 0; found: 51.87; H, 2.25; N, 0.

Synthesis of 1S′

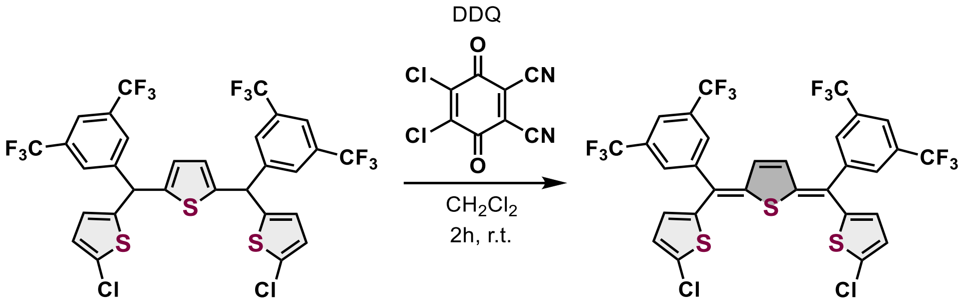

To a 100 mL flask, **3S′** (393.4 mg, 0.5112 mmol, 1 eq), dichloromethane (51 mL) and DDQ (127.4 mg, 0.5612 mmol, 1.098 eq) were added and stirred under room temperature at ambient condition for 120 minutes. After reaction, purification by column chromatography (Silica gel 60N, hexane) afforded an orange solid of **1S′** as a mixture of three geometric isomers (35.9 mg, 9%). The ^{13}C NMR spectrum was further complicated by the existence of diastereomer and splitting of signals due to coupling with ^{19}F nuclei. Due to these factors, the ^{13}C NMR data for **1S′** are not provided here, and the spectrum is presented in Figure S18. ^{1}H NMR (500 MHz,$(CD_3)_2CO$) δ = 8.21–8.03 (m, Ar), 7.62 (s, *EE*), 7.44 (d, *J* = 6.2 Hz, *EZ*), 7.13 (d, *J* = 4.2 Hz, *ZZ*), 7.09 (d, *J* = 4.1 Hz, *EE*), 7.07 (d, *J* = 4.1 Hz, *EZ*), 7.02 (d, *J* = 4.2 Hz, *EZ*), 6.96 (d, *J* = 3.9 Hz, *EE*), 6.95 (d, *J* = 4.0 Hz, *EZ*), 6.90 (d, *J* = 4.2 Hz, *ZZ*), 6.81 (d, *J* = 6.2 Hz, *EZ*), 6.73 (d, *J* = 4.2 Hz, *EZ*), 6.62 (s, *ZZ*); HRMS(ESI): *m/z* calcd for $C_{30}H_{12}Cl_2F_{12}S_3$: 765.9281 ([M]$^+$), found 765.9284; E.A.: calcd. for $C_{30}H_{12}Cl_2F_{12}S_3$: C, 46.95; H, 1.58; N, 0; 47.07; H, 1.79; N, 0.

Measurement of Isomer Ratio at Thermal Equilibrium

The equilibrium ratios of the geometric isomers at different temperatures were determined as follows. NMR samples were prepared in sealed tubes with deuterated solvent ($CDCl_3$, [D$_6$]acetone, or [D$_2$]tetrachloroethane). These samples were heated in a metal bath at the designated temperature outside the NMR instrument. At each temperature, samples were maintained until reaching equilibrium, which was confirmed by taking multiple measurements over time until no further changes in isomer ratios were observed. Prior to NMR analysis, the heated samples were rapidly cooled to room temperature and promptly measured at room temperature (25 °C).

This rapid cooling approach is viable because, as demonstrated in Figures S1 and S2, the interconversion between isomers at room temperature is negligibly slow, with no significant changes observed over several days. The "frozen" equilibrium state thus accurately reflects the isomer distribution at the elevated temperature.

For solvent-dependent studies reported in Table S1 (acetonitrile and cyclohexane), a modified procedure was employed. The compounds were dissolved in the respective non-deuterated solvents and heated in sealed test tubes to 55 °C until equilibrium was reached. After equilibration, the samples were cooled to low temperature, and the solvent was completely removed under reduced pressure. The residue was then immediately redissolved in $CDCl_3$ for NMR analysis to determine the isomer ratios. This approach ensured that the equilibrium distributions established in the original solvents were preserved during the measurement process.

2. Additional spectroscopic data

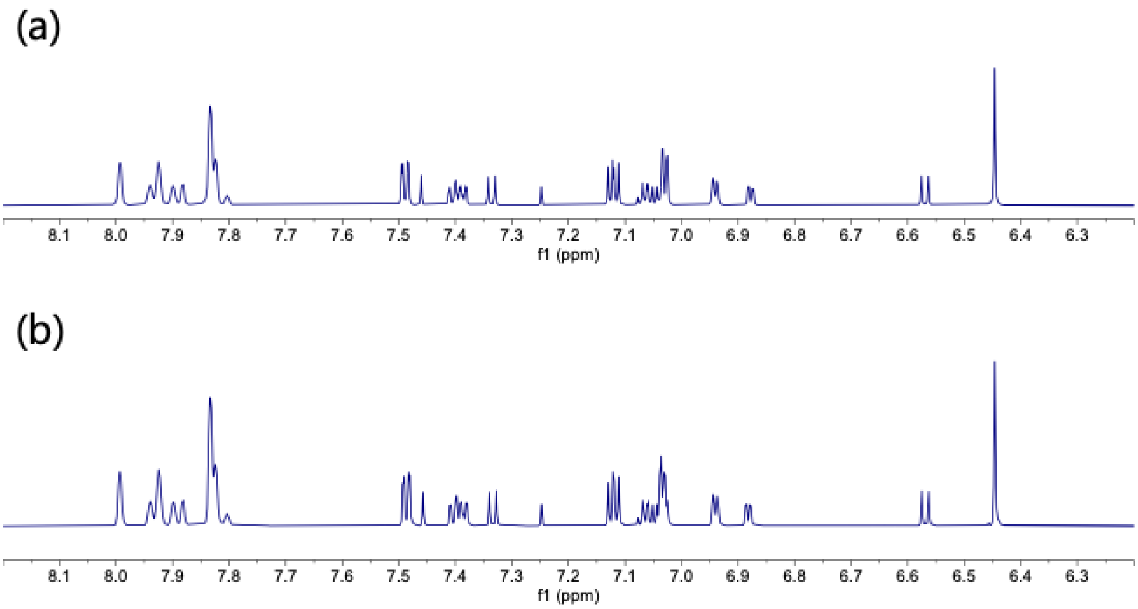

Figure S1. ^{1}H NMR spectra (500 MHz, $CDCl_3$, r.t.) of geometric isomer mixtures of **1S** (non-equilibrium state) indicating negligible interconversion between three isomers at room temperature. (a) Immediately after sample dissolution. (b) After 10 hours at room temperature.

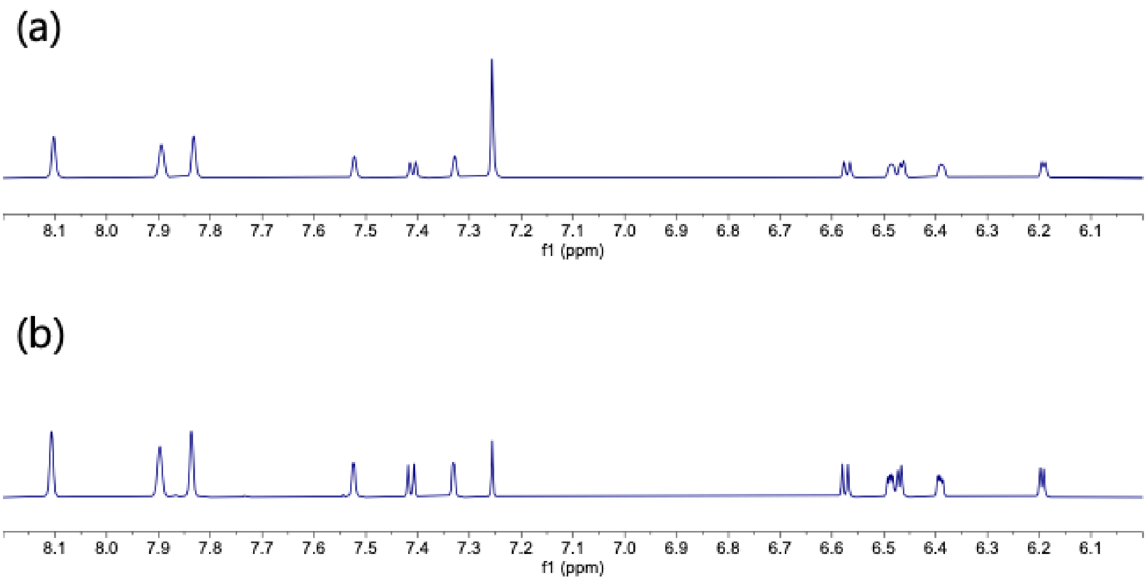

Figure S2. ^{1}H NMR spectra (500 MHz, $CDCl_3$, r.t.) of pure *EZ*-**1O** indicating negligible interconversion between three isomers. (a) Immediately after sample dissolution. (b) After 3 days at room temperature.

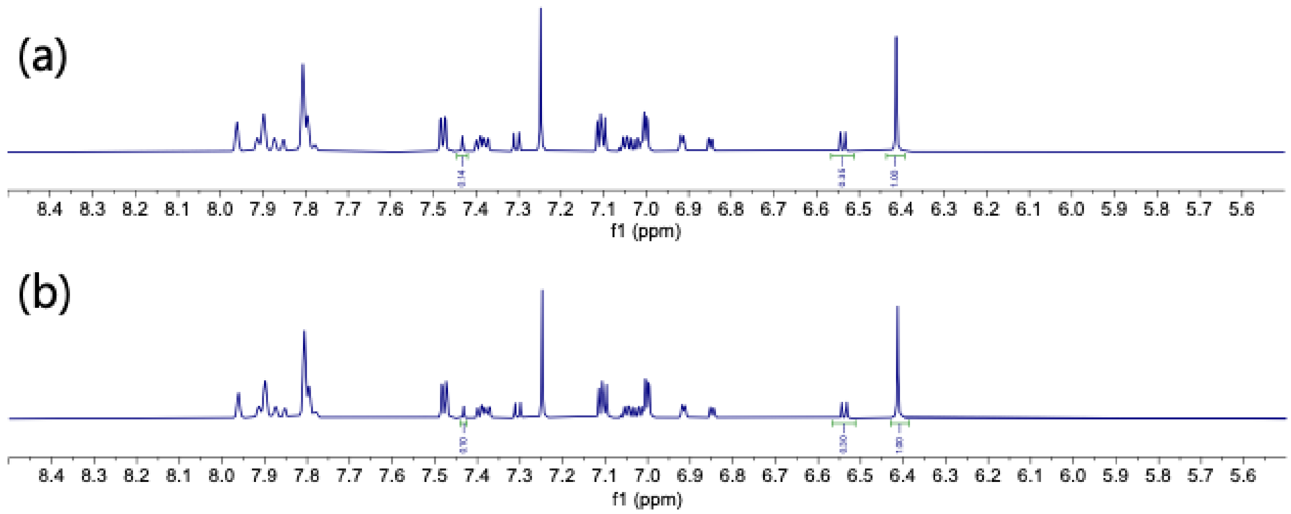

Figure S3. ^{1}H NMR spectra ($CDCl_3$, r.t.) showing the process of reaching thermal equilibrium at 55°C for the three geometric isomers of **1S**. Samples were analyzed following the equilibration procedure described in the Experimental Section. (a) 0 hours, (b) after 24 hours at 55°C (thermal equilibrium state).

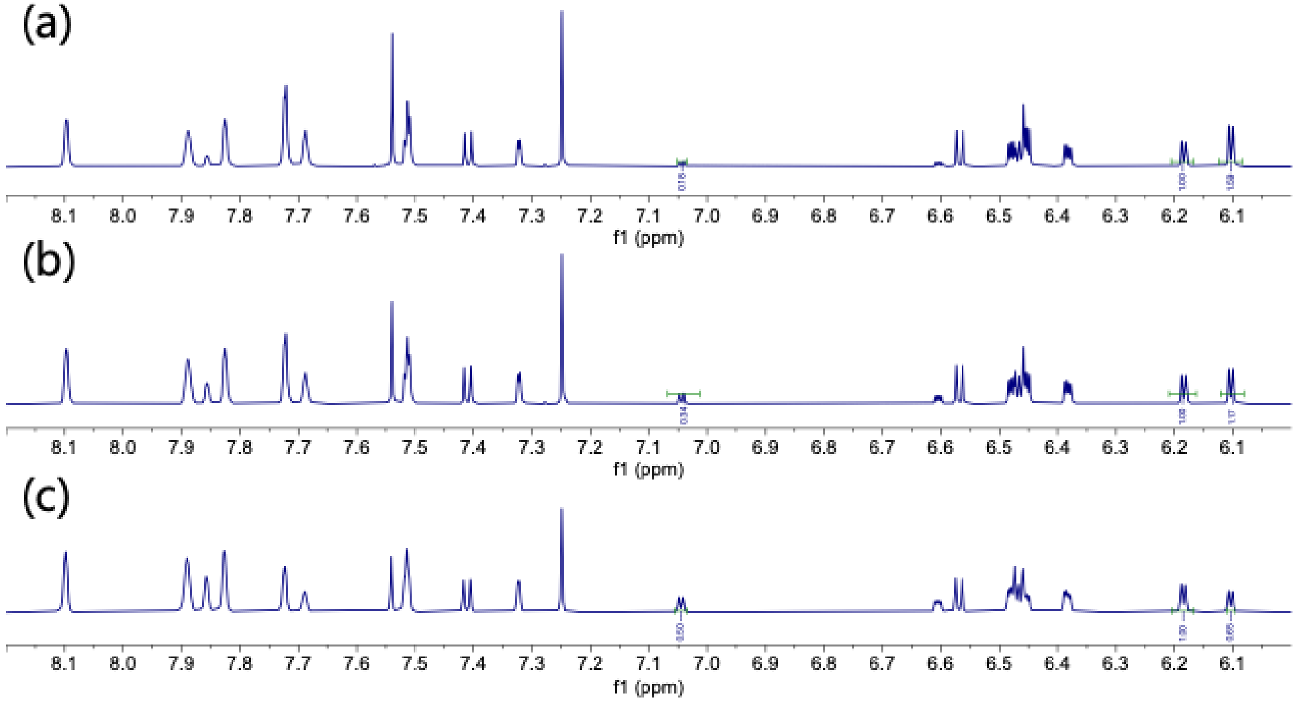

Figure S4. ^{1}H NMR spectra ($CDCl_3$, r.t.) showing the process of reaching thermal equilibrium at 55°C for the three geometric isomers of **1O**. Samples were analyzed following the equilibration procedure described in the Experimental Section. (a) 0 hours, (b) after 24 hours at 55°C, (c) after 119 hours at 55°C (thermal equilibrium state).

Table S1. The ratio of geometrical isomer at thermal equilibrium state in each solvent (55 °C).

Solvent	**1O**			**1S**		
	ZZ	*EZ*(+*ZE*)	*EE*	*ZZ*	*EZ*(+*ZE*)	*EE*
[D_6]acetone	22	56	21	63	32	5
$CDCl_3$	16	64	20	58	35	6
acetonitrile	18	59	23	64	32	4
cyclohexane	18	65	17	56	37	7

*Determined according to the procedure described in the Experimental Section.

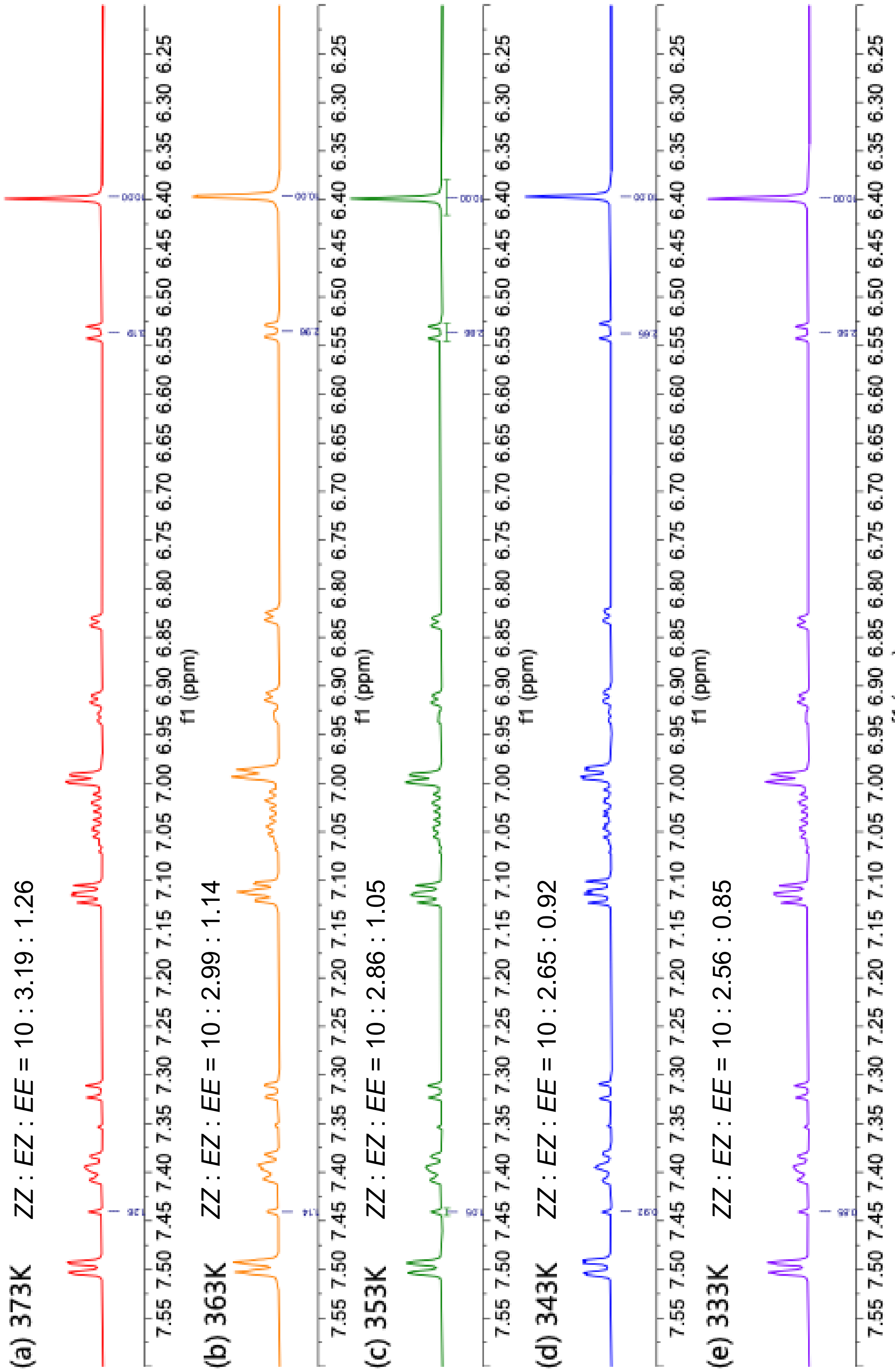

Figure S5. ^{1}H NMR spectra (500 MHz, r.t.) of **1S** in [D_2]tetrachloroethane showing the equilibrium distribution of three geometric isomers at different temperatures.

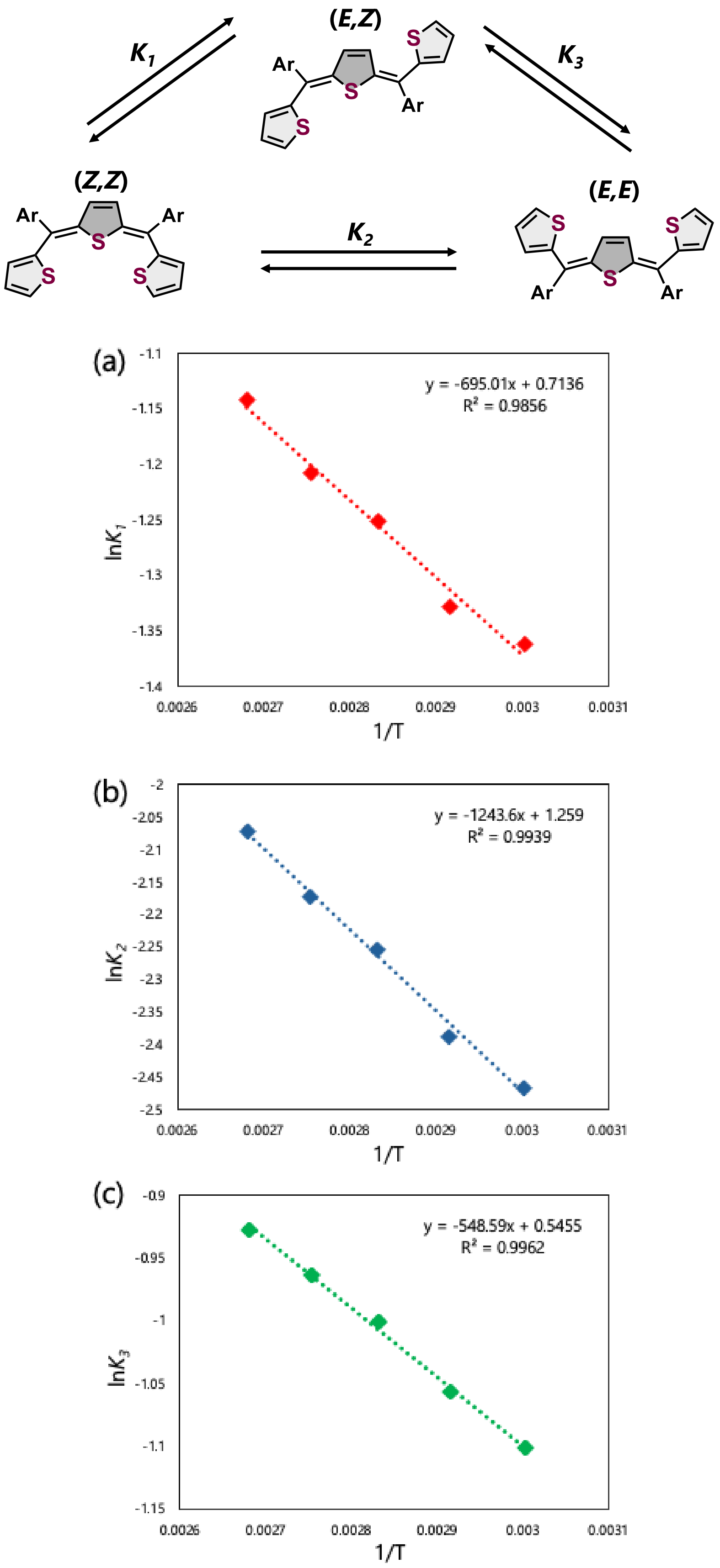

Figure S6. Van't Hoff plot for each equilibrium reaction of **1S**. (a) *ZZ*-*ZE*, (b) *ZZ*-*EE*, (c) *ZE*-*EE*.

Table S2. The thermodynamic parameters for each equilibrium reaction of **1S**.

	ΔH kcal/mol	ΔS cal/mol K	$\Delta G_{328\ \mathrm{K}}$ kcal/mol
ZZ-EZ	1.38±0.10	1.41±0.27	0.92±0.12
ZZ-EE	2.47±0.11	2.51±0.32	1.65±0.14
EZ-EE	1.09±0.04	1.09±0.11	0.74±0.05

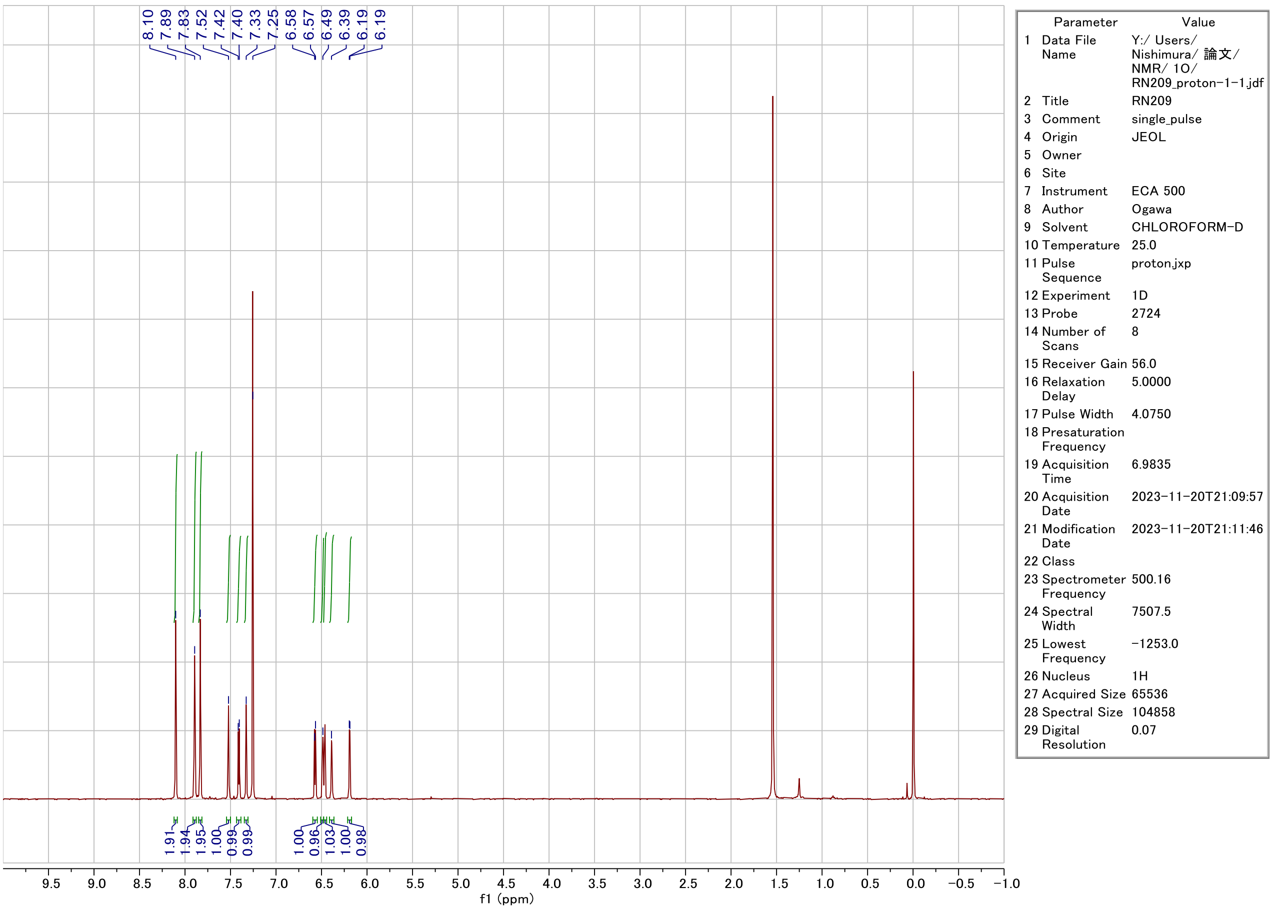

Parameter		Value
1	Data File Name	Y:/ Users/ Nishimura/ 論文/ NMR/ 1O/ RN209_proton-1-1.jdf
2	Title	RN209
3	Comment	single_pulse
4	Origin	JEOL
5	Owner	
6	Site	
7	Instrument	ECA 500
8	Author	Ogawa
9	Solvent	CHLOROFORM-D
10	Temperature	25.0
11	Pulse Sequence	proton.jxp
12	Experiment	1D
13	Probe	2724
14	Number of Scans	8
15	Receiver Gain	56.0
16	Relaxation Delay	5.0000
17	Pulse Width	4.0750
18	Presaturation Frequency	
19	Acquisition Time	6.9835
20	Acquisition Date	2023-11-20T21:09:57
21	Modification Date	2023-11-20T21:11:46
22	Class	
23	Spectrometer Frequency	500.16
24	Spectral Width	7507.5
25	Lowest Frequency	-1253.0
26	Nucleus	1H
27	Acquired Size	65536
28	Spectral Size	104858
29	Digital Resolution	0.07

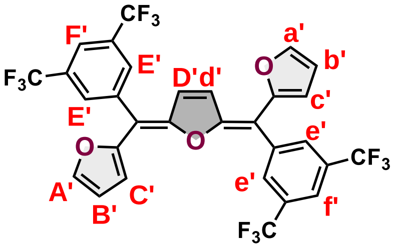

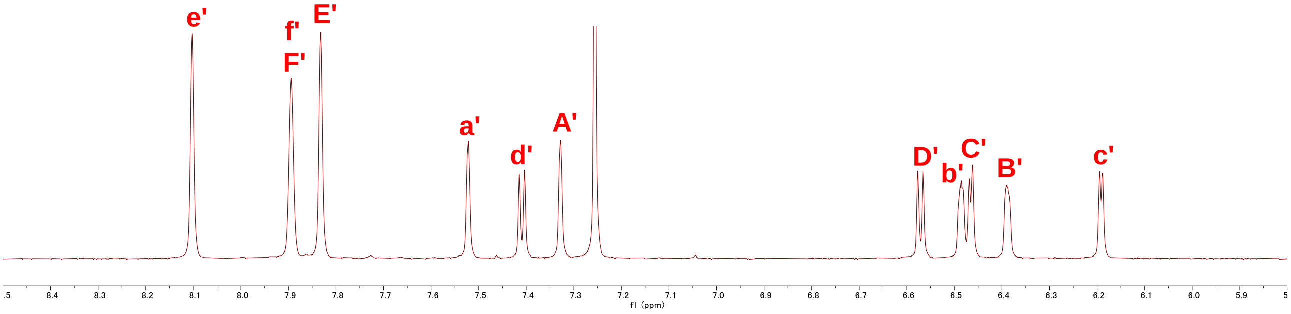

Figure S7. Full and expended ^{1}H NMR spectrum (500 MHz, $CDCl_3$, r.t.) of *EZ*-**1O**.

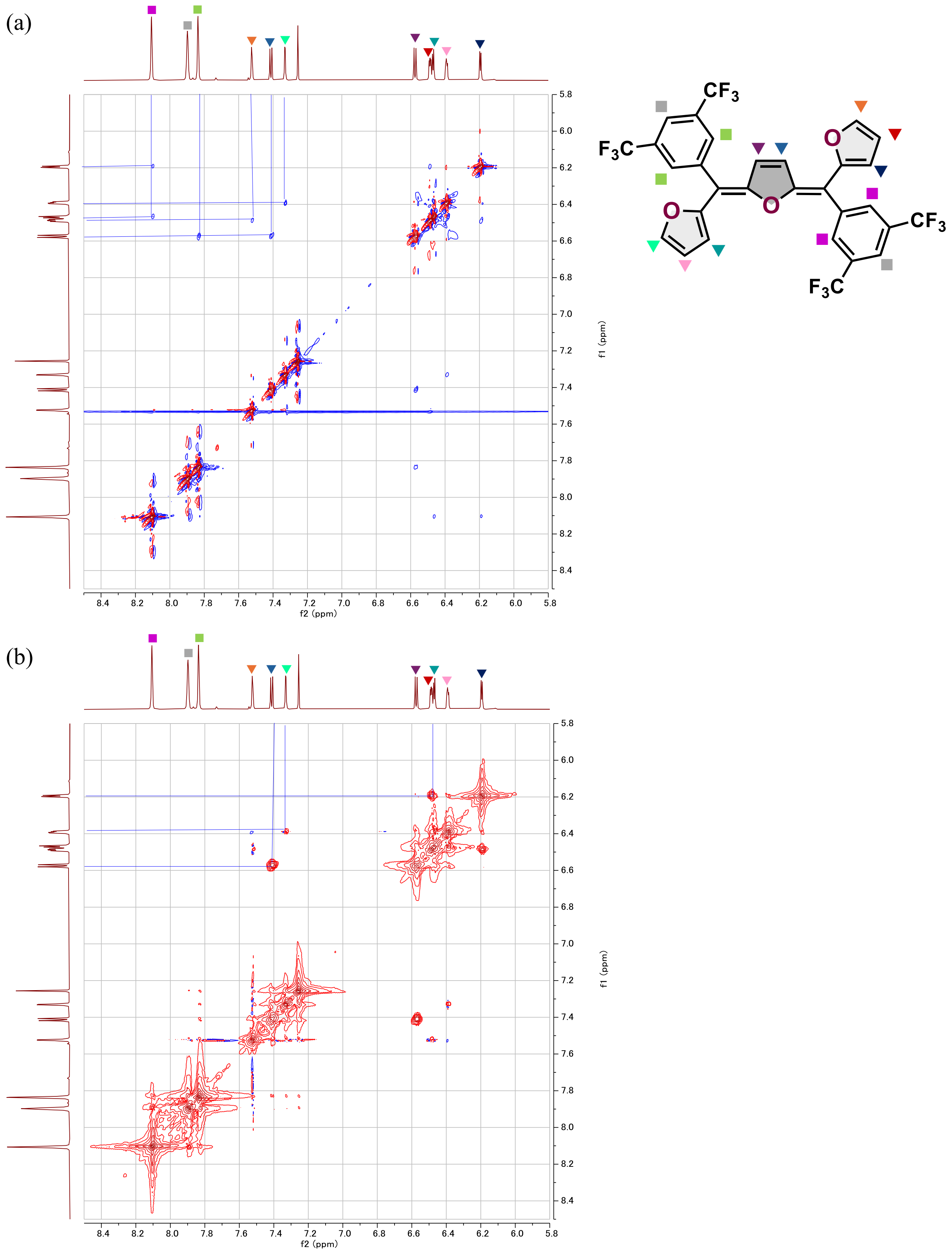

Figure S8. (a) NOESY and (b) COSY spectra (500 MHz, $CDCl_3$) of *EZ*-**1O**.

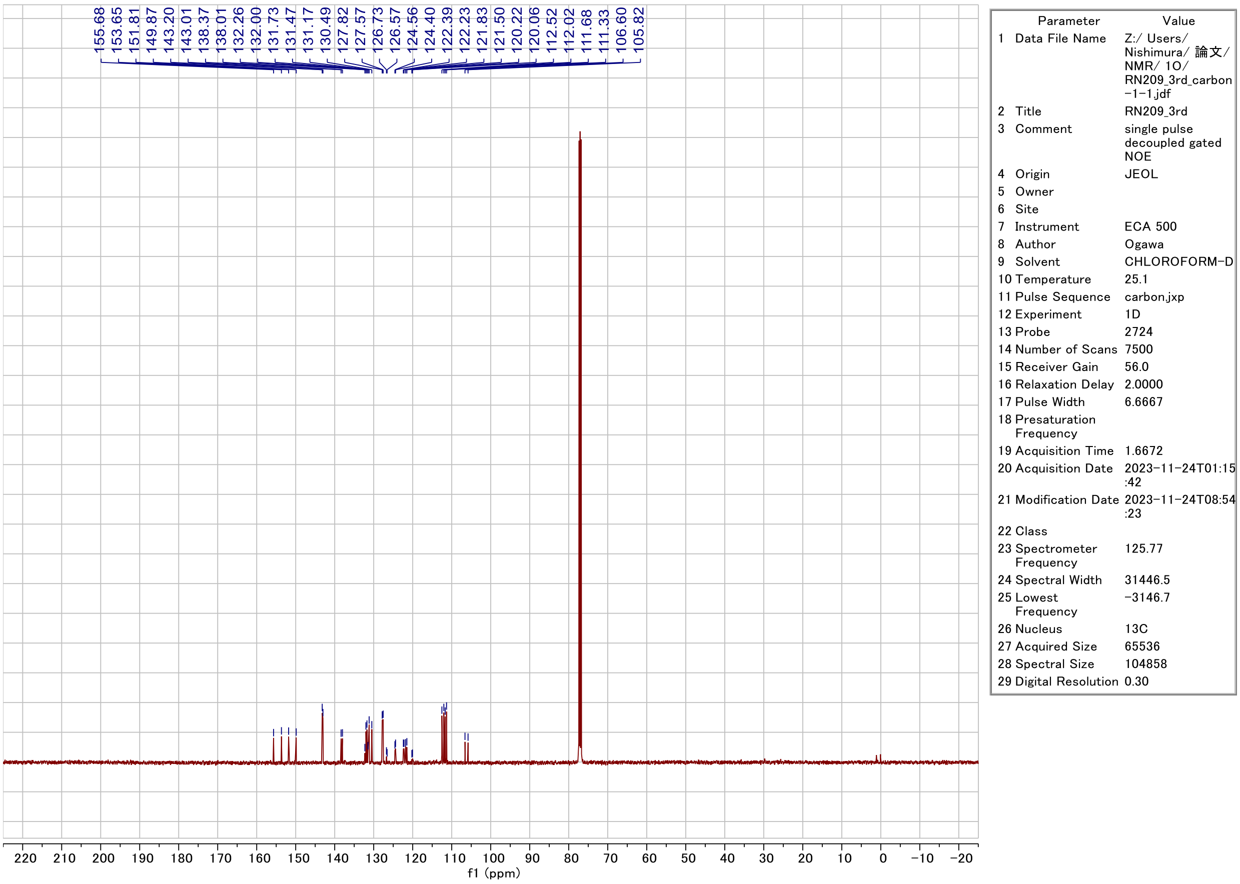

Figure S9. ^{13}C NMR spectrum (125 MHz, $CDCl_3$) of *EZ*-**1O**.

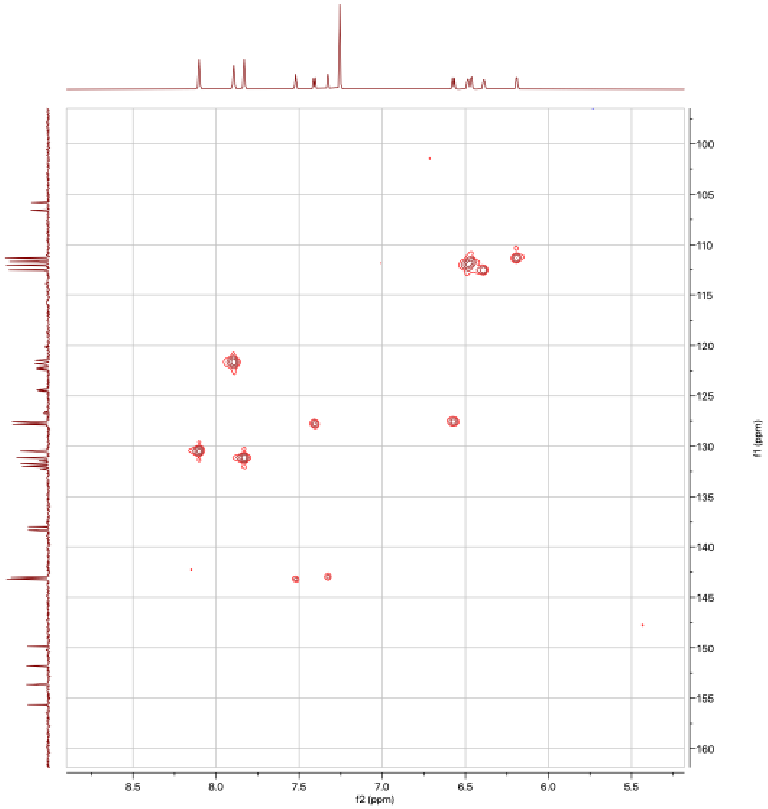

Figure S10. HSQC spectrum ($CDCl_3$) of *EZ*-**1O**.

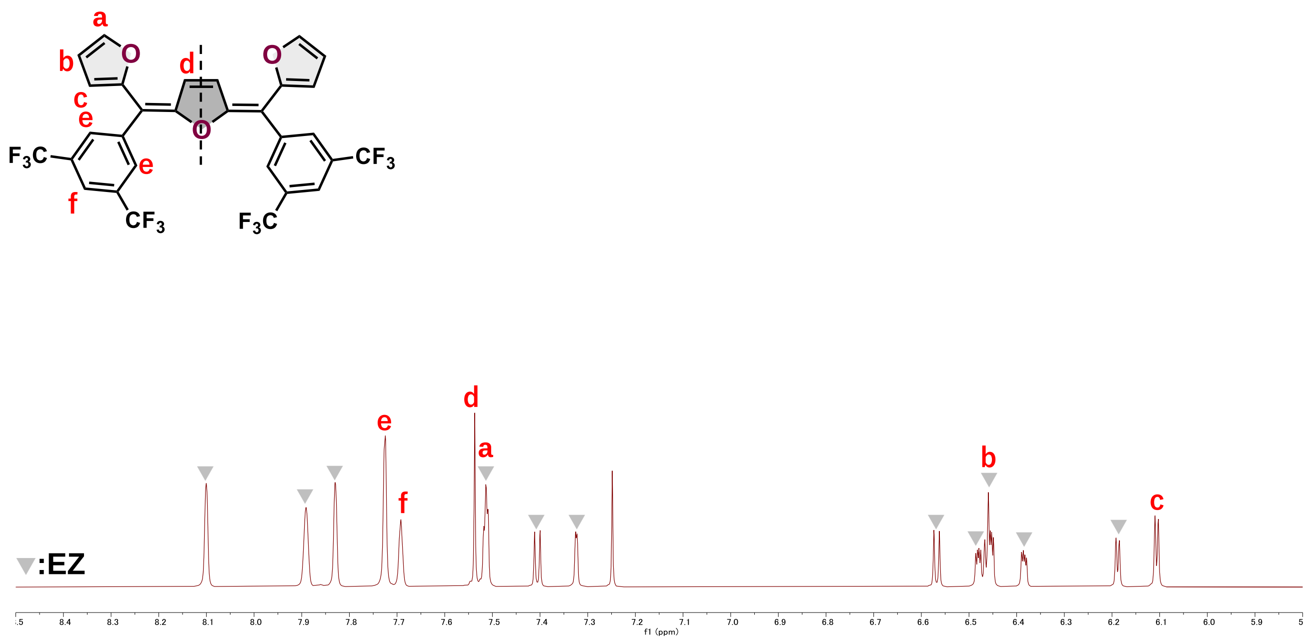

Figure S11. ^{1}H NMR (500 MHz, $CDCl_3$, r.t.) spectrum of geometric isomer mixtures of **1O**.

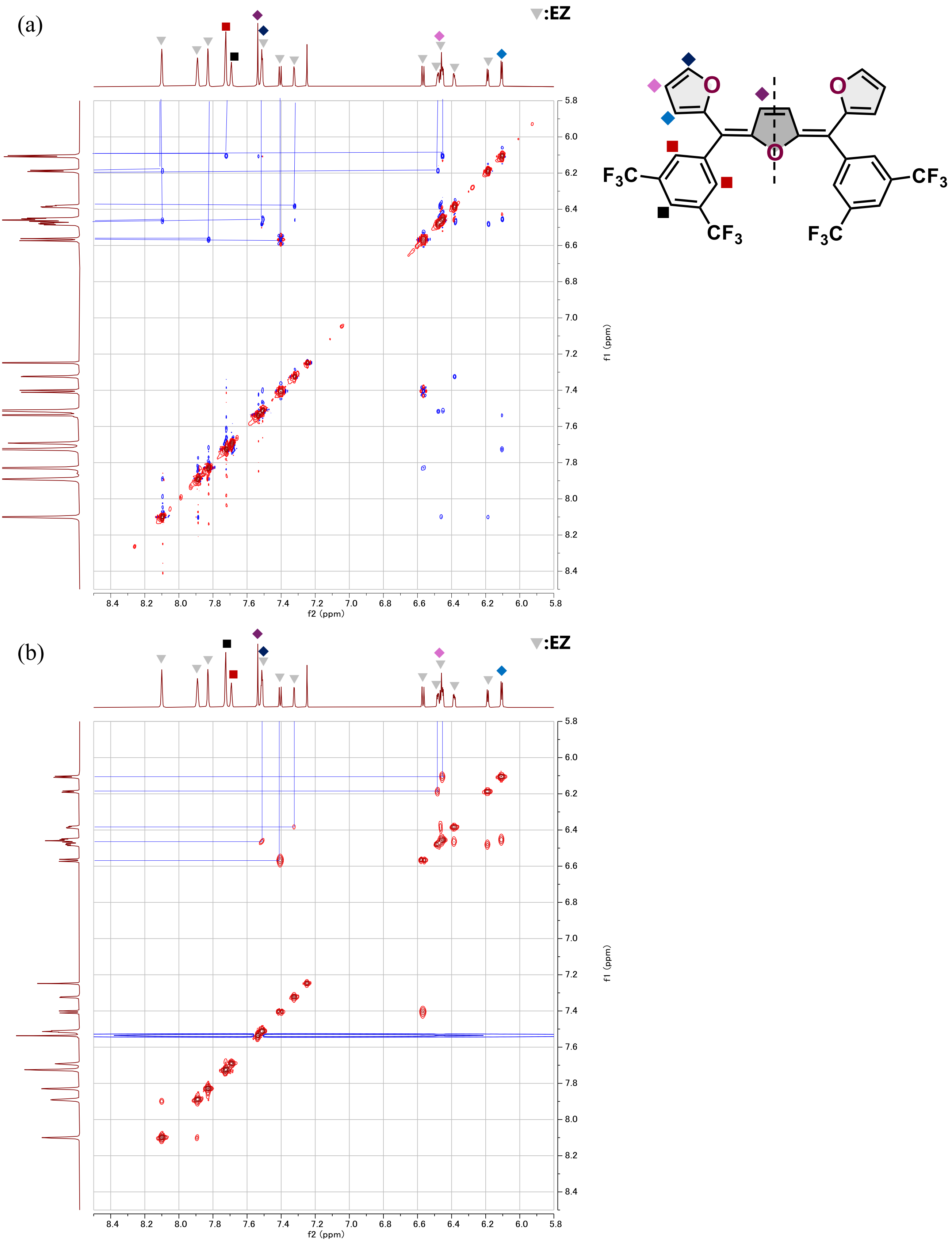

Figure S12. (a) NOESY and (b) COSY spectra (500 MHz, $CDCl_3$) of geometric isomer mixtures of **1O**.

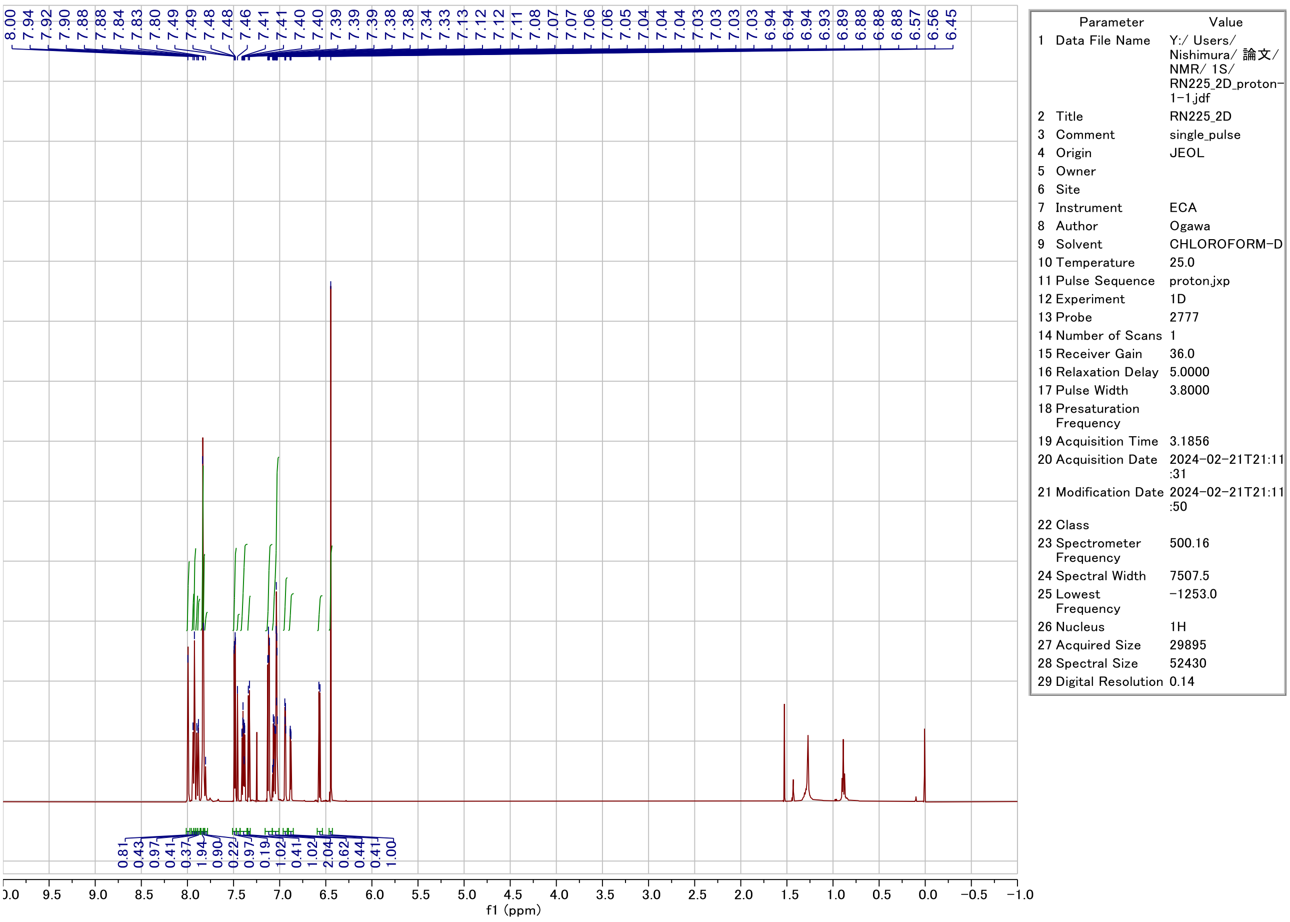

	Parameter	Value
1	Data File Name	Y:/ Users/ Nishimura/ 論文/ NMR/ 1S/ RN225_2D_proton-1-1.jdf
2	Title	RN225_2D
3	Comment	single_pulse
4	Origin	JEOL
5	Owner	
6	Site	
7	Instrument	ECA
8	Author	Ogawa
9	Solvent	CHLOROFORM-D
10	Temperature	25.0
11	Pulse Sequence	proton.jxp
12	Experiment	1D
13	Probe	2777
14	Number of Scans	1
15	Receiver Gain	36.0
16	Relaxation Delay	5.0000
17	Pulse Width	3.8000
18	Presaturation Frequency	
19	Acquisition Time	3.1856
20	Acquisition Date	2024-02-21T21:11:31
21	Modification Date	2024-02-21T21:11:50
22	Class	
23	Spectrometer Frequency	500.16
24	Spectral Width	7507.5
25	Lowest Frequency	−1253.0
26	Nucleus	1H
27	Acquired Size	29895
28	Spectral Size	52430
29	Digital Resolution	0.14

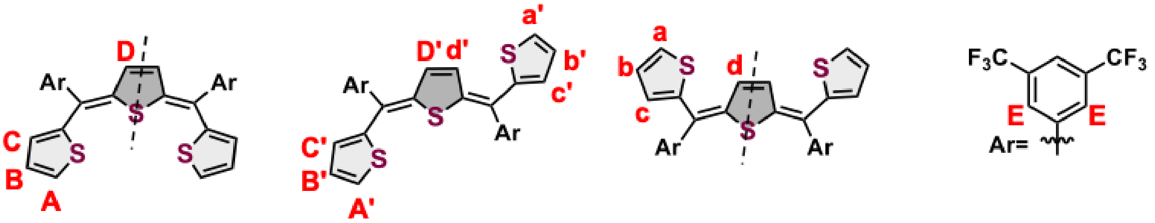

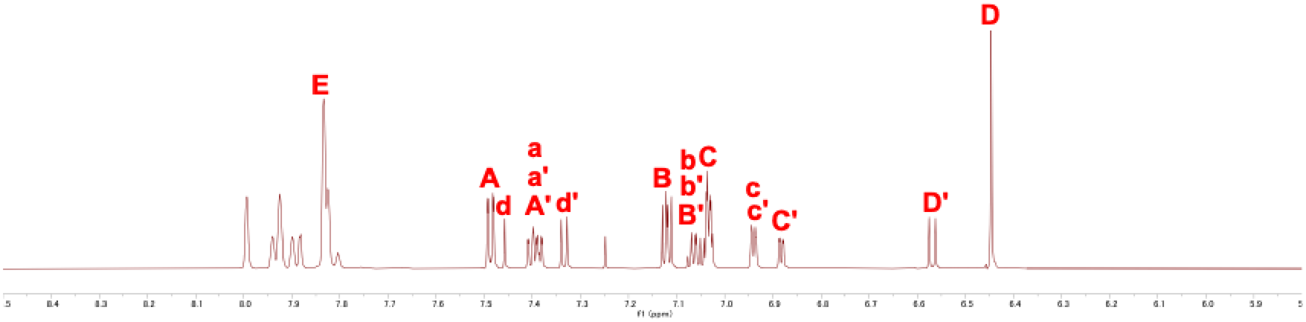

Figure S13. Full and expended ^{1}H NMR spectrum (500 MHz, $CDCl_3$, r.t.) of geometric isomer mixtures of **1S**.

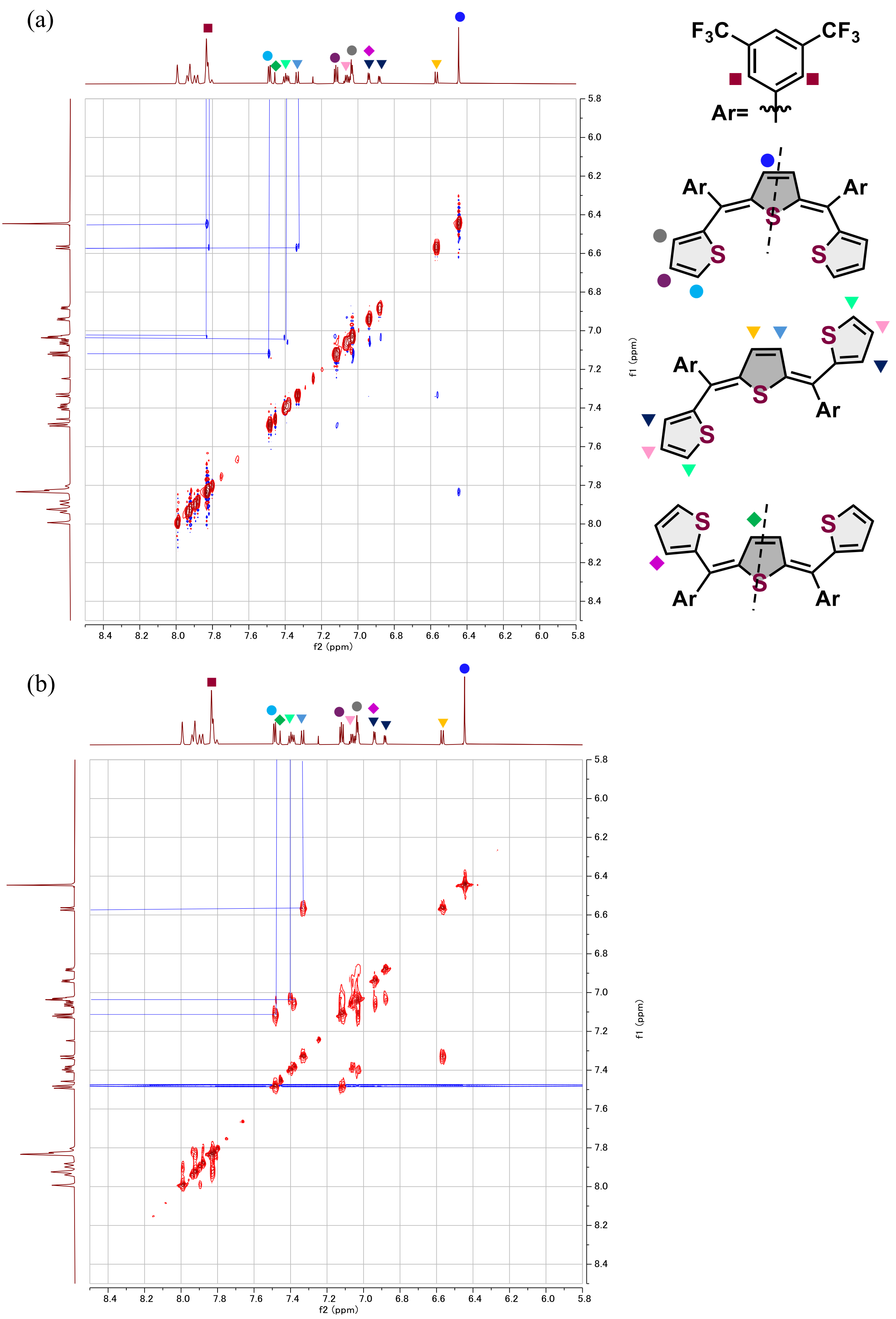

Figure S14. (a) NOESY and (b) COSY spectra (500 MHz, $CDCl_3$) of **1S** (a mixture of the diastereomers).

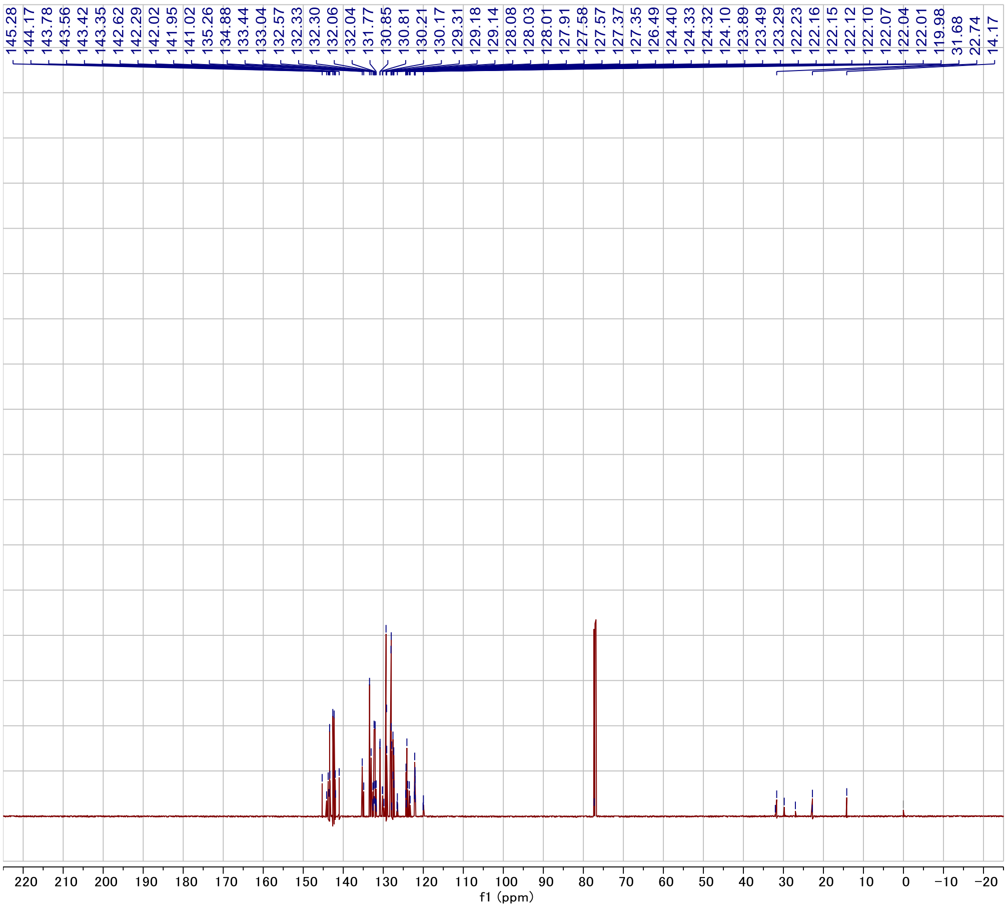

	Parameter	Value
1	Data File Name	Z:/ Users/ Nishimura/ 論文/ NMR/ 1S/ RN225_2D_carbon−1−1.jdf
2	Title	RN225_2D
3	Comment	single pulse decoupled gated NOE
4	Origin	JEOL
5	Owner	
6	Site	
7	Instrument	ECA
8	Author	Ogawa
9	Solvent	CHLOROFORM−D
10	Temperature	25.0
11	Pulse Sequence	carbon.jxp
12	Experiment	1D
13	Probe	2777
14	Number of Scans	4500
15	Receiver Gain	56.0
16	Relaxation Delay	2.2858
17	Pulse Width	3.8667
18	Presaturation Frequency	
19	Acquisition Time	0.7142
20	Acquisition Date	2024−02−22T05:02:21
21	Modification Date	2024−02−22T08:47:37
22	Class	
23	Spectrometer Frequency	125.77
24	Spectral Width	31446.5
25	Lowest Frequency	−3146.7
26	Nucleus	13C
27	Acquired Size	28074
28	Spectral Size	52430
29	Digital Resolution	0.60

Figure S15. ^{13}C NMR spectrum (125 MHz, $CDCl_3$) of geometric isomer mixtures of **1S**.

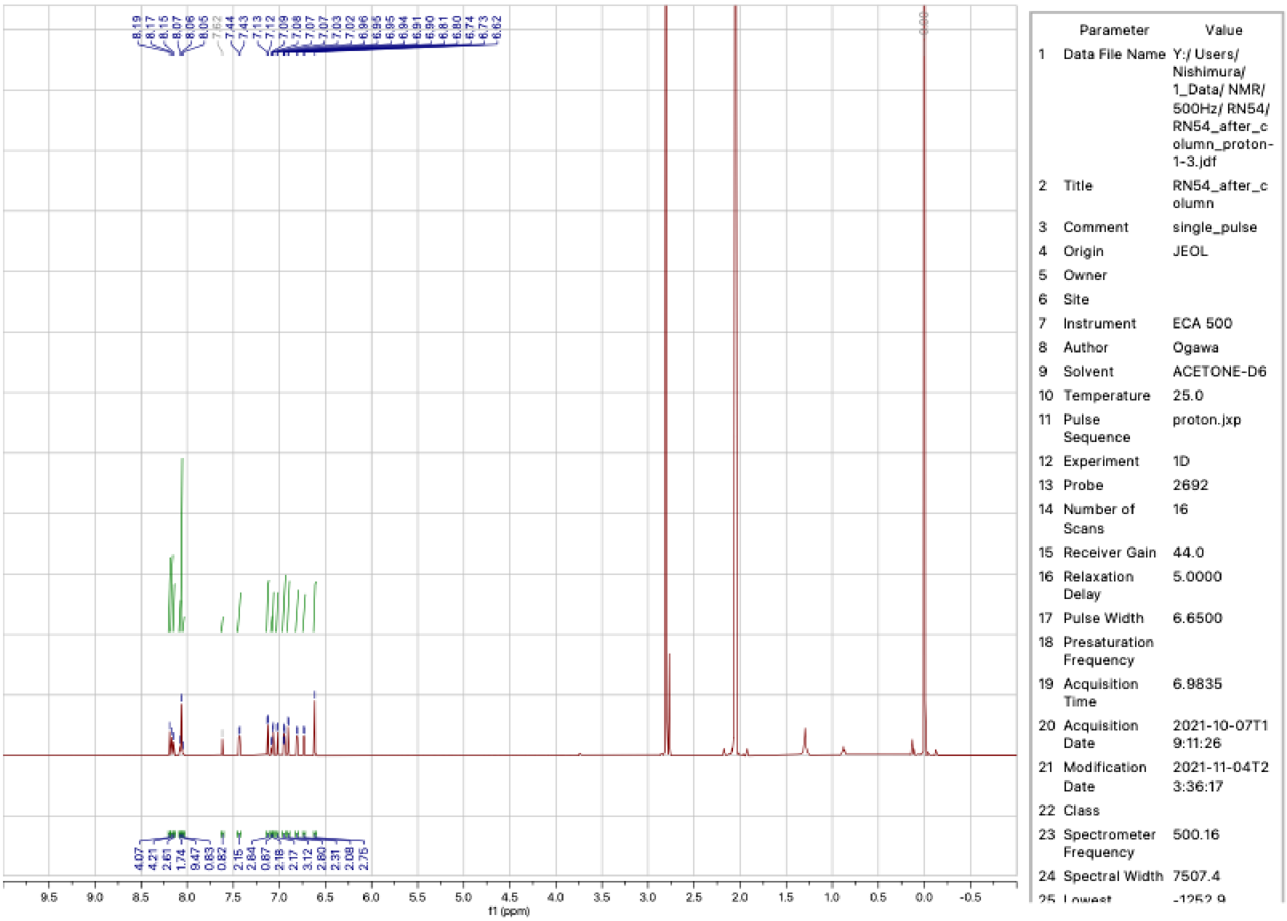

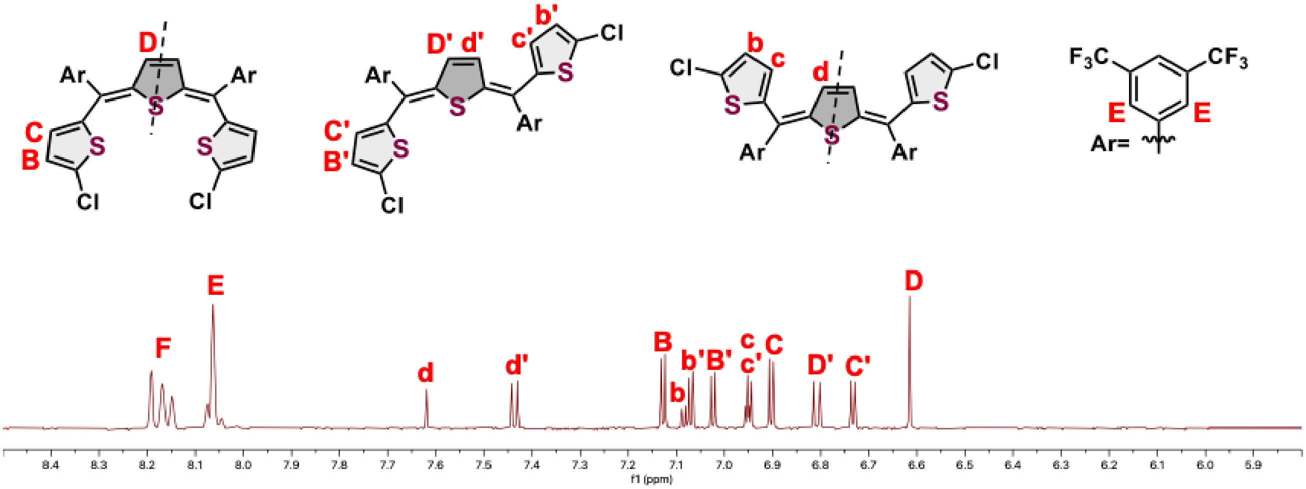

Figure S16. Full and expended ¹H NMR (500 MHz, $(CD_3)_2CO$, r.t.) spectrum of geometric isomer mixtures of **1S′**.

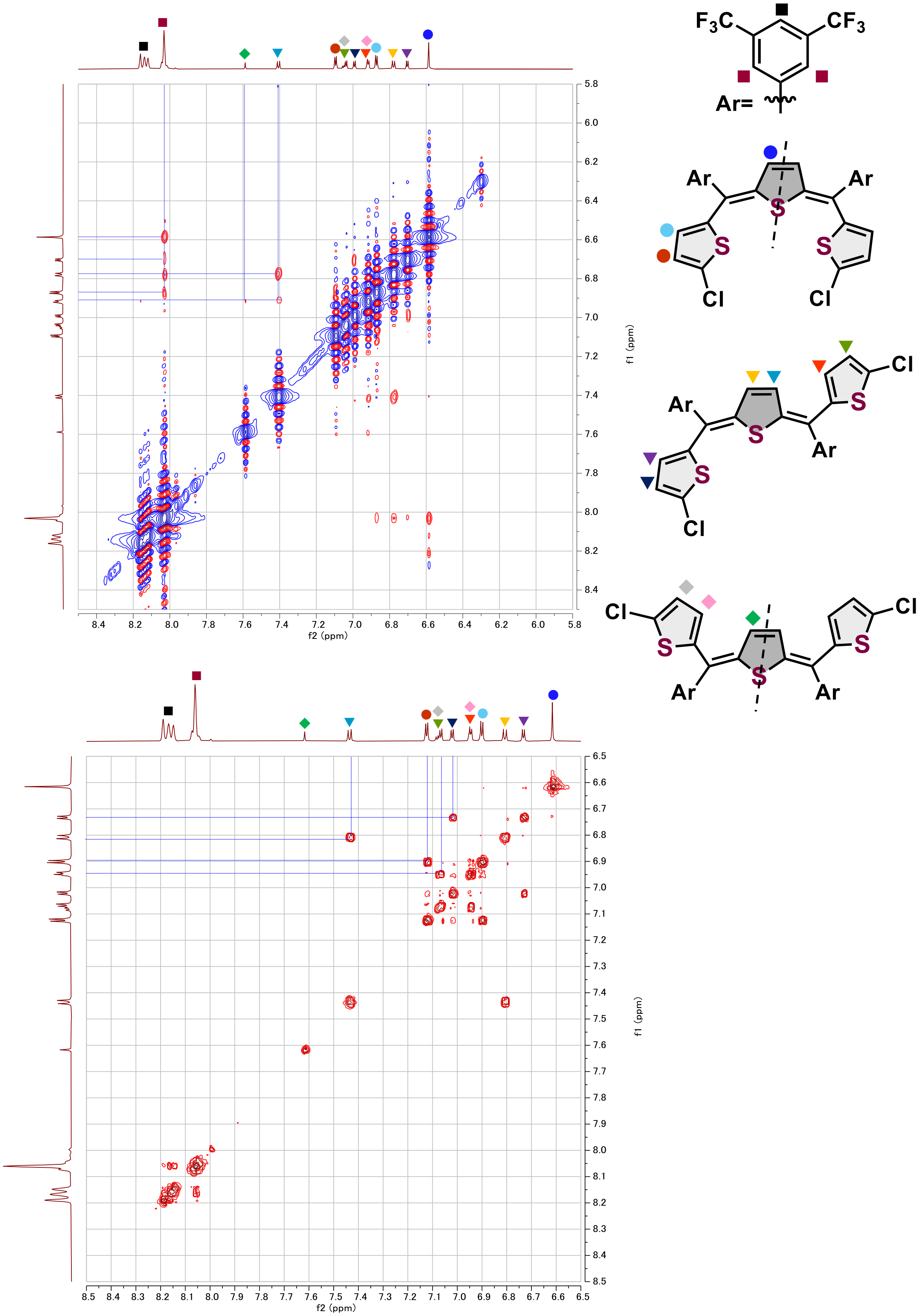

Figure S17. (a) NOESY and (b) COSY spectra (500 MHz, $(CD_3)_2CO$) of **1S′** (a mixture of the diastereomers).

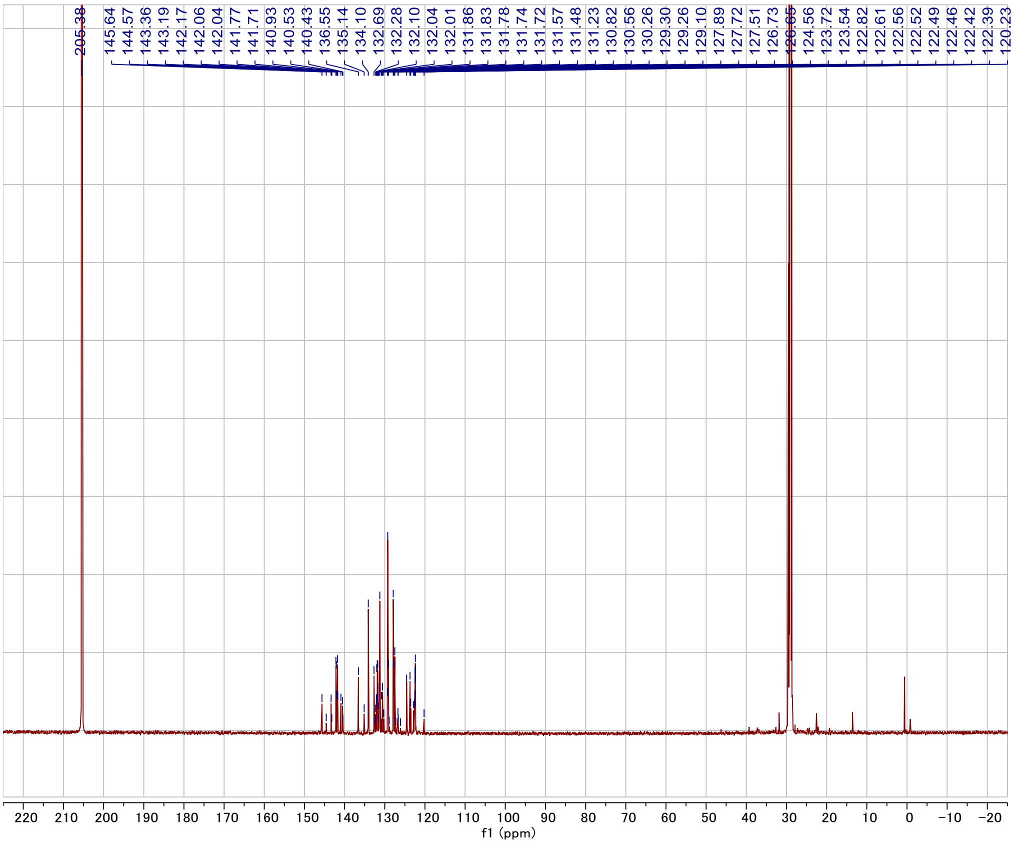

	Parameter	Value
1	Data File Name	Z:/ Users/ Nishimura/ 1_Data/ NMR/ 500Hz/ RN54/ RN54_carbon-1-1.jdf
2	Title	RN54
3	Comment	single pulse decoupled gated NOE
4	Origin	JEOL
5	Owner	
6	Site	
7	Instrument	ECA 500
8	Author	Ogawa
9	Solvent	ACETONE-D6
10	Temperature	17.3
11	Pulse Sequence	carbon.jxp
12	Experiment	1D
13	Probe	2692
14	Number of Scans	12000
15	Receiver Gain	60.0
16	Relaxation Delay	2.0000
17	Pulse Width	3.7667
18	Presaturation Frequency	
19	Acquisition Time	0.8336
20	Acquisition Date	2021-10-25T23:13:05
21	Modification Date	2021-10-26T08:40:03
22	Class	
23	Spectrometer Frequency	125.77
24	Spectral Width	31446.5
25	Lowest Frequency	−3146.7
26	Nucleus	13C
27	Acquired Size	32768
28	Spectral Size	52430
29	Digital Resolution	0.60

Figure S18. ^{13}C NMR spectrum (125 MHz, $(CD_3)_2CO$) of geometric isomer mixtures of **1S′**.

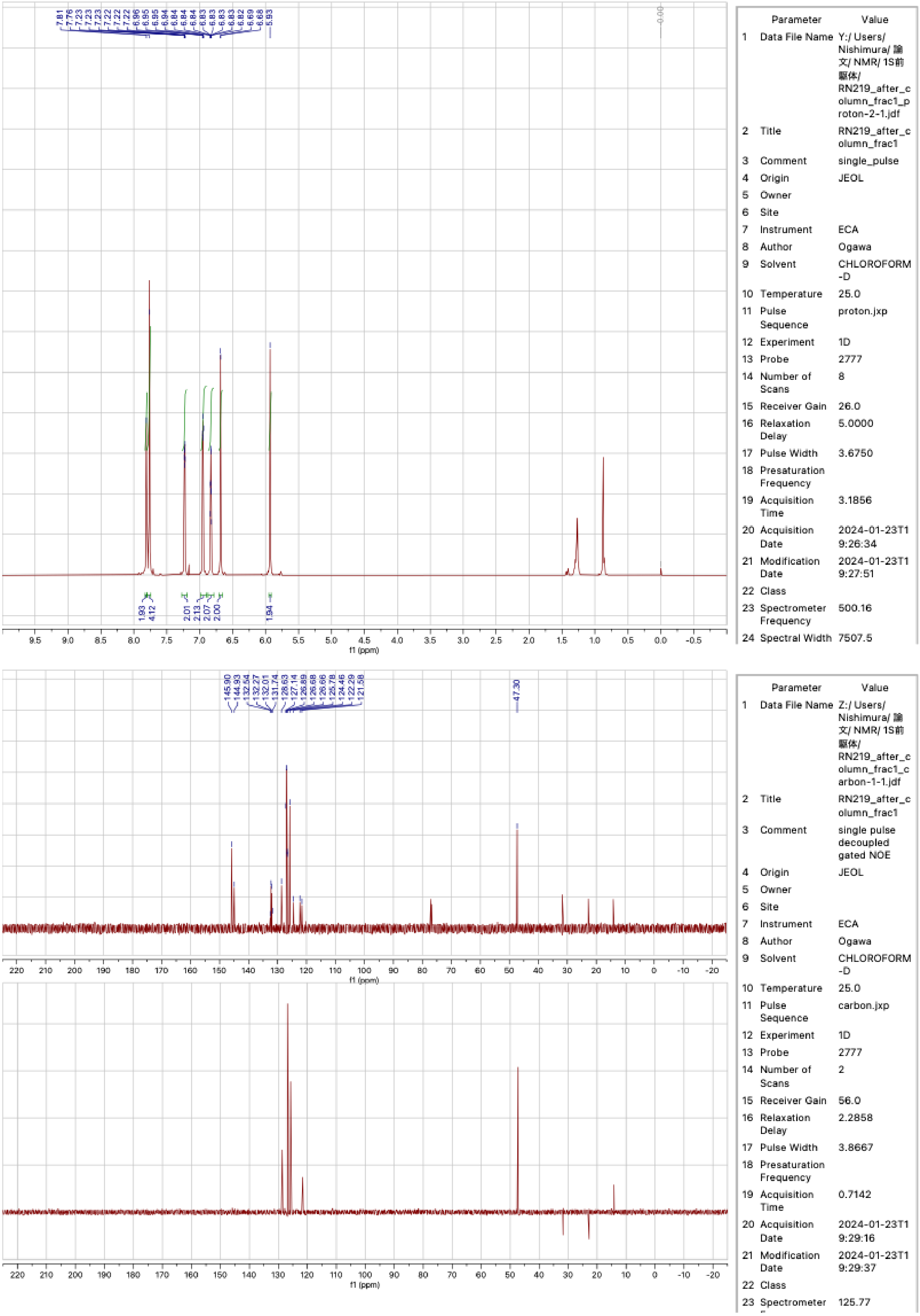

Figure S19. (a) ^{1}H and (b) ^{13}C NMR spectra of **3S**.

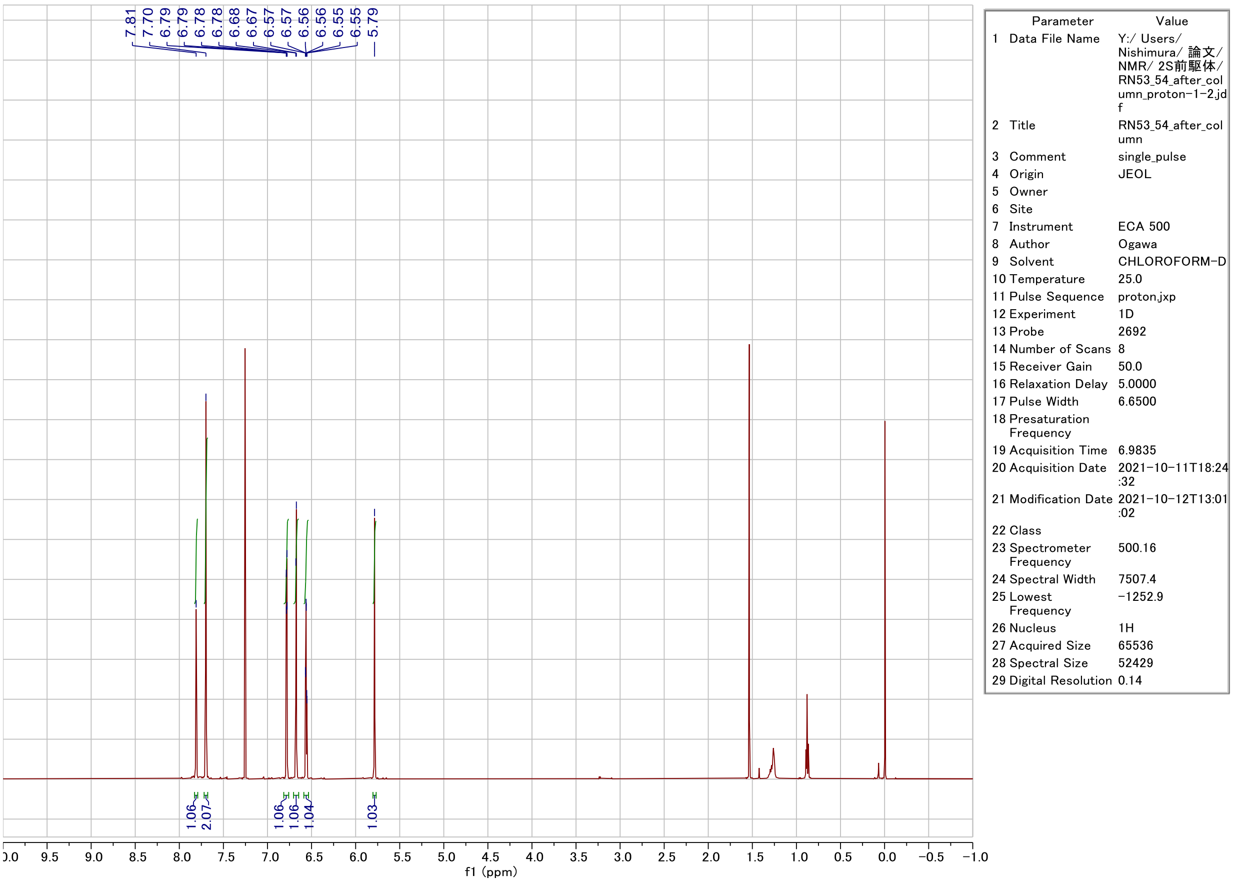

	Parameter	Value
1	Data File Name	Y:/ Users/ Nishimura/ 論文/ NMR/ 2S前駆体/ RN53_54_after_column_proton-1-2.jdf
2	Title	RN53_54_after_column
3	Comment	single_pulse
4	Origin	JEOL
5	Owner	
6	Site	
7	Instrument	ECA 500
8	Author	Ogawa
9	Solvent	CHLOROFORM-D
10	Temperature	25.0
11	Pulse Sequence	proton.jxp
12	Experiment	1D
13	Probe	2692
14	Number of Scans	8
15	Receiver Gain	50.0
16	Relaxation Delay	5.0000
17	Pulse Width	6.6500
18	Presaturation Frequency	
19	Acquisition Time	6.9835
20	Acquisition Date	2021-10-11T18:24:32
21	Modification Date	2021-10-12T13:01:02
22	Class	
23	Spectrometer Frequency	500.16
24	Spectral Width	7507.4
25	Lowest Frequency	-1252.9
26	Nucleus	1H
27	Acquired Size	65536
28	Spectral Size	52429
29	Digital Resolution	0.14

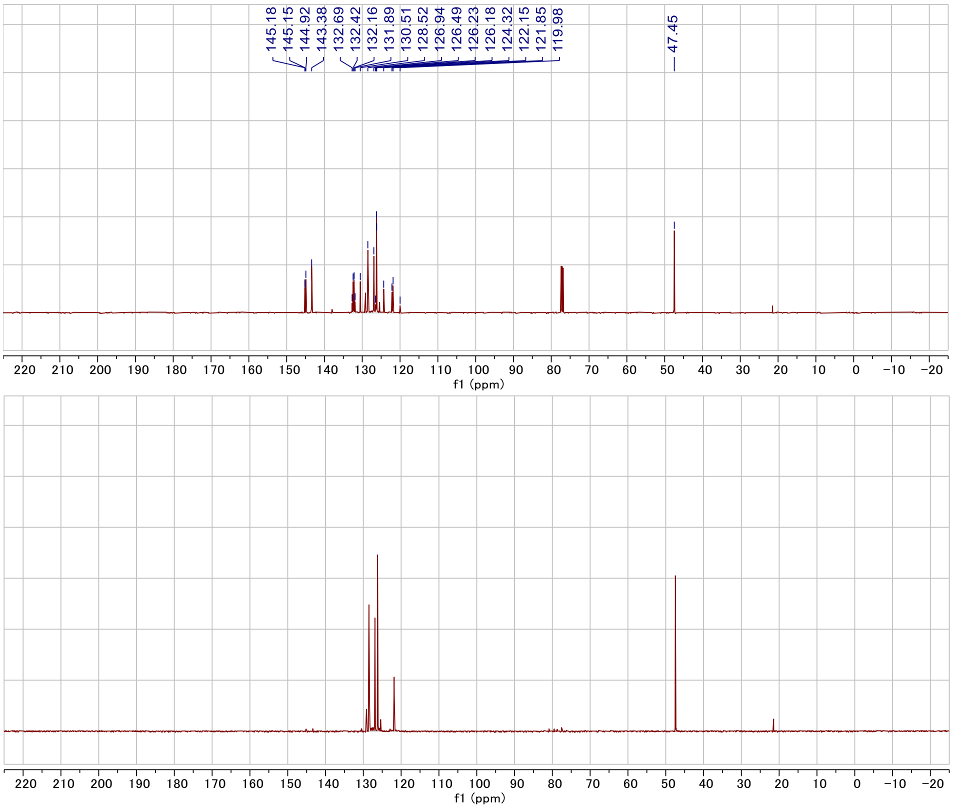

	Parameter	Value
1	Data File Name	Z:/ Users/ Nishimura/ 論文/ NMR/ 2S前駆体/ RN53_crude_13C_carbon-1-2.jdf
2	Title	RN53_crude_13C
3	Comment	single pulse decoupled gated NOE
4	Origin	JEOL
5	Owner	
6	Site	
7	Instrument	ECA 500
8	Author	Ogawa
9	Solvent	CHLOROFORM-D
10	Temperature	25.0
11	Pulse Sequence	carbon.jxp
12	Experiment	1D
13	Probe	2692
14	Number of Scans	302
15	Receiver Gain	58.0
16	Relaxation Delay	2.0000
17	Pulse Width	3.7667
18	Presaturation Frequency	
19	Acquisition Time	0.8336
20	Acquisition Date	2021-10-06T14:35:48
21	Modification Date	2021-10-06T19:49:50
22	Class	
23	Spectrometer Frequency	125.77
24	Spectral Width	31444.9
25	Lowest Frequency	-3145.9
26	Nucleus	13C
27	Acquired Size	32768
28	Spectral Size	26214
29	Digital Resolution	1.20

Figure S20. (a) ^{1}H and (b) ^{13}C NMR spectra of **3S′**.

3. X-Ray crystal structure determinations

Orange block crystals of *ZZ*-**1S′** were grown by slow diffusion of methanol vapor into its chloroform solution. Orange prism crystals of *EZ*-**1S′** were grown by slow diffusion of hexane vapor into its chloroform solution. Orange block crystals of *EE*-**1O** were grown by concentrating the filtrate after removal of *EZ*-**1O** by recrystallization from hot EtOH, dissolving the concentrate in acetonitrile, and then cooling the solution. Single-crystal X-ray diffraction data were collected on a Rigaku XtaLAB Synergy Custom diffractometer using multilayer mirror monochromated Mo-$K\alpha$ radiation (λ = 0.71075 Å) by the ω scan mode. The crystal was cooled by a stream of cold N_2 gas. Collection, indexing, peak integration, cell refinement, and scaling of the diffraction data were performed using CrysAlisPro 1.171.41.122a software (Rigaku OD, 2021). The data were corrected for Lorentz and polarization effects, and empirical absorption correction was applied. The structures were solved using SHELXT[71] programs and refined by full-matrix least-squares calculations on F^2 (SHELXL).[72,73] All non-hydrogen atoms were modeled anisotropically. All hydrogen atoms were placed in idealized positions and refined using a riding model [U_{iso}(H) = 1.2U_{eq}(C)].

The crystallographic data are summarized in Table S3. Deposition Numbers 2388514 (for *ZZ*-**1S′**), 2388513 (for *EZ*-**1S′**), 2388515 (for *EE*-**1O**) contains the supplementary crystallographic data for this paper. These data are provided free of charge by the joint Cambridge Crystallographic Data Centre and Fachinformationszentrum Karlsruhe Access Structures service.

Table S3. Crystallographic data.

	ZZ-**1S′**	*EZ*-**1S′**	*EE*-**1O**
CCDC No.	2388514	2388513	2388515
empirical formula	$C_{30}H_{12}Cl_2F_{12}S_3$	$C_{30}H_{12}Cl_2F_{12}S_3$	$C_{30}H_{14}F_{12}O_3$
formula weight	767.48	767.48	650.41
temperature / K	153	173	153
crystal system	Triclinic	Monoclinic	Monoclinic
space group	P-1	$P2_1/c$	$P2_1/n$
a / Å	14.6820(2)	9.6942(5)	13.4186(4)
b / Å	15.3230(2)	27.0442(13)	14.9062(4)
c / Å	15.6043(3)	11.6194(6)	26.8766(9)
α / deg	96.4510(10)	90	90
β / deg	115.719(2)	94.367(5)	97.049(3)
γ / deg	90.0350(10)	90	90
V / Å^3	3137.95(10)	3037.4(3)	5335.2(3)
Z	4	4	8
D (calcd) / g cm^{-3}	1.625	1.678	1.619
μ / mm^{-1}	0.500	0.517	0.160
F(000)	1528	1528	2608
crystal size / mm^3	0.200×0.200×0.150	0.300×0.040×0.030	0.550×0.400×0.150
θ range for data collection / deg	1.460 to 30.526	1.912 to 30.608	1.527 to 30.756
index ranges	$-20 \le h \le 20$, $-21 \le k \le 21$, $-21 \le l \le 21$	$-12 \le h \le 9$, $-36 \le k \le 26$, $-14 \le l \le 11$	$-16 \le h \le 16$, $-20 \le k \le 20$, $-34 \le l \le 36$
reflections collected	85032	26280	63810
independent reflections	16539 [R(int) = 0.0590]	7500 [R(int) = 0.0762]	13607 [R(int) = 0.0308]
completeness	100.0%	100.0%	100.0%
data / restraints / parameters	16539 / 79 / 943	7500 / 24 / 483	13607 / 606 / 923
GOF on F^2	1.282	1.060	1.049
final R indices [$I > 2\sigma(I)$]^a	R_1 = 0.0553, wR_2 = 0.1725	R_1 = 0.0603, wR_2 = 0.1469	R_1 = 0.0506, wR_2 = 0.1331
R indices (all data)^a	R_1 = 0. 0695, wR_2 = 0.1832	R_1 = 0.0959, wR_2 = 0.1658	R_1 = 0. 0678, wR_2 = 0.1442
largest diff. peak and hole / e·A^{-3}	1.446 and -0.652	0.496 and -0.620	0.646 and -0.578

^{a} $R_1 = \sum||F_o|-|F_c||/\sum|F_o|$, $wR_2 = [\sum[w(F_o^2-F_c^2)^2]/\sum w(F_o^2)^2]^{1/2}$.

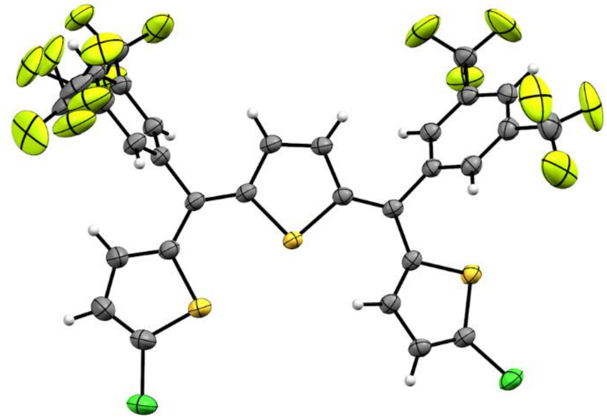

Figure S21. The crystal structure of *ZZ*-**1S′** (Molecule A) (C = gray, H = white, S = orange, F = yellow, and Cl = green). Thermal ellipsoids are drawn at 50% probability level.

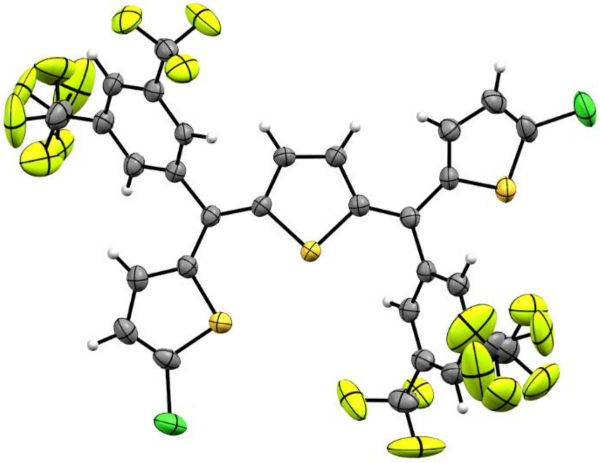

Figure S22. The crystal structure of *EZ*-**1S′** (C = gray, H = white, S = orange, F = yellow, and Cl = green). Thermal ellipsoids are drawn at 50% probability level.

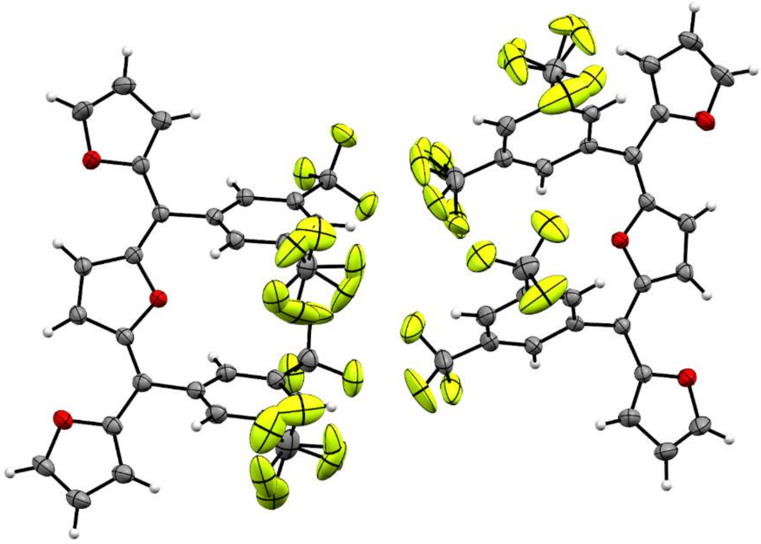

Figure S23. The crystal structure of *EE*-**1O** (C = gray, H = white, O = red, F = yellow). Thermal ellipsoids are drawn at 50% probability level.

4. Theoretical calculations

The conformational/configurational spaces of **1O** and **1S** were sampled using the Global Optimizer Algorithm (GOAT),[48] included in ORCA 6.0.1.[49,50] at the semiempirical GFN1-xTB[51,52] calculation level with 12 kcal/mol threshold of Gibbs free energy (at 55 °C). The resulting ensembles were then further optimized using CENSO 1.2.0[47] with ORCA 5.0.4.[49,74] part 1 (prescreening), and part2 (optimization) with default settings were used, and part 2 was performed at the r^{2}SCAN-3c[53] level. The solvent effect for chloroform solution was included by employing the implicit solvation model. Coordinates of the obtained ensembles can be found in the other SI files.

The proportions of each geometric isomer presented in Table 1 were calculated as follows. While CENSO automatically outputs the Gibbs energy for each conformer along with their population distribution (Boltzmann weight %), the default settings of GOAT do not provide the degeneracy of each conformer (for example, it does not output the *ZE* conformer as a degenerate identical conformation for a given *EZ* conformer), rendering these Boltzmann weights inaccurate. Therefore, we assigned degeneracy values based on molecular symmetry: conformers with mirror symmetry were assigned a value of 1 (e.g., *syn,syn-ZZ*, *anti,anti-EE*), while those without mirror symmetry were assigned a value of 2 (e.g., *EZ*(=*ZE*), *syn,anti-ZZ*). The proportion of each geometric isomer was then calculated according to the standard formula using these degeneracy values and the calculated Gibbs free energies. Coordinates of the obtained conformer ensembles can be found in the other SI files.

$$x_{ZZ} = \sum_{i \in ZZ} g_i P_i \Big/ \sum_i g_i P_i , \qquad x_{EZ,ZE} = \sum_{i \in EZ,ZE} g_i P_i \Big/ \sum_i g_i P_i , \qquad x_{EE} = \sum_{i \in EE} g_i P_i \Big/ \sum_i g_i P_i$$

x_{ZZ}: mole fraction of ZZ, g_i: degeneracy of conformer i, $P_i = \exp(-G_i/kT)$,
G_i: Gibbs free energy of confomer i, k: Boltzmann constant, T: temperature (328 K)

Theoretical analyses of noncovalent interactions for *syn,syn-ZZ*-**1S** were carried out using the Gaussian 16 program package (Revision C.01).[75] Its geometry was optimized using the DFT method at the B3LYP-D3BJ/def2-TZVP level. To confirm that the optimized geometries were not at the saddle but stable points, frequency calculations were performed. Natural bond orbital (NBO) analysis was calculated by NBO7.0 programs[64] using geometry-optimized structures.

The noncovalent interactions (NCI),[56] electron localization function (ELF),[54] and quantum theory of atoms in molecules (QTAIM)[57–60] analyses were conducted through Multiwfn package[76] using the wave function obtained by above calculation. The NCI RDG isosurface (s = 0.5 a.u.) was visualized using VMD.[77] The scatter graph was visualized using Gnuplot 6.0.

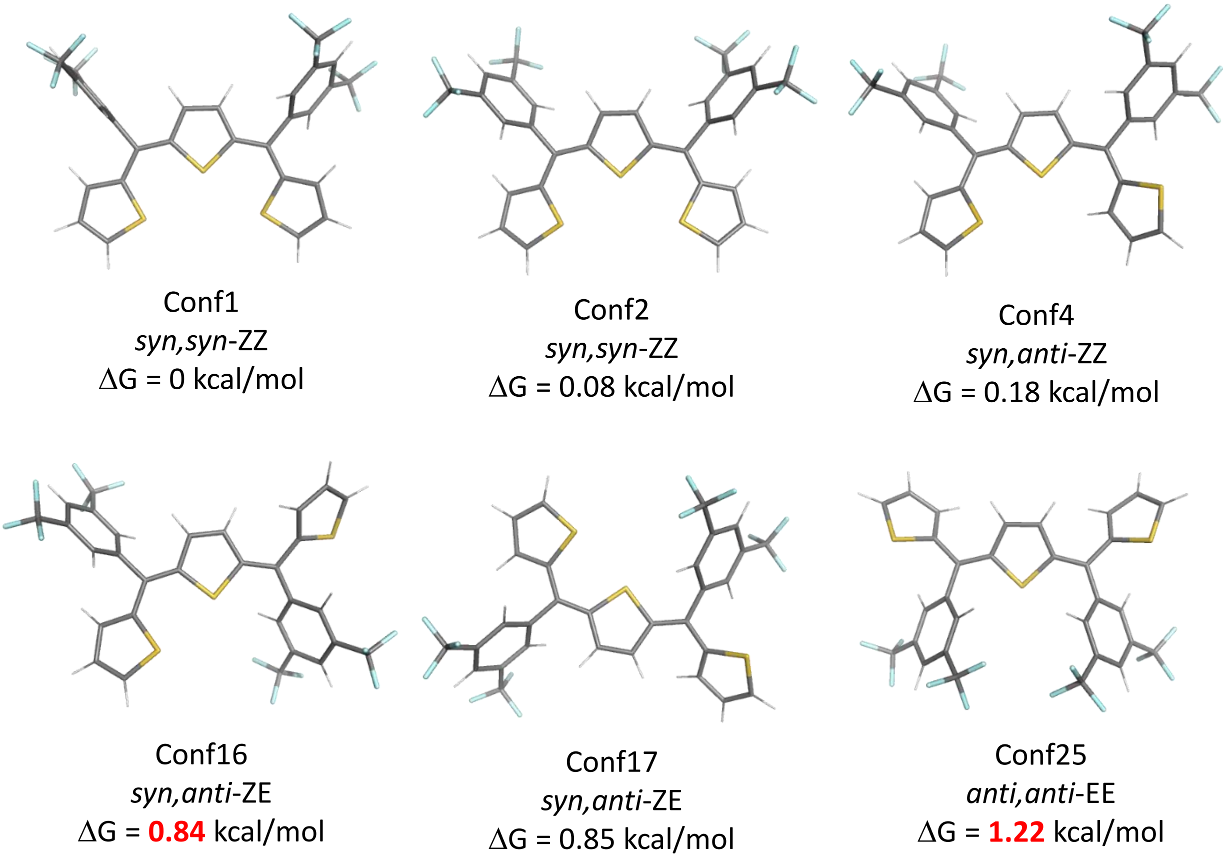

Figure S24. Selected conformers of **1S** obtained by GOAT/CENSO.

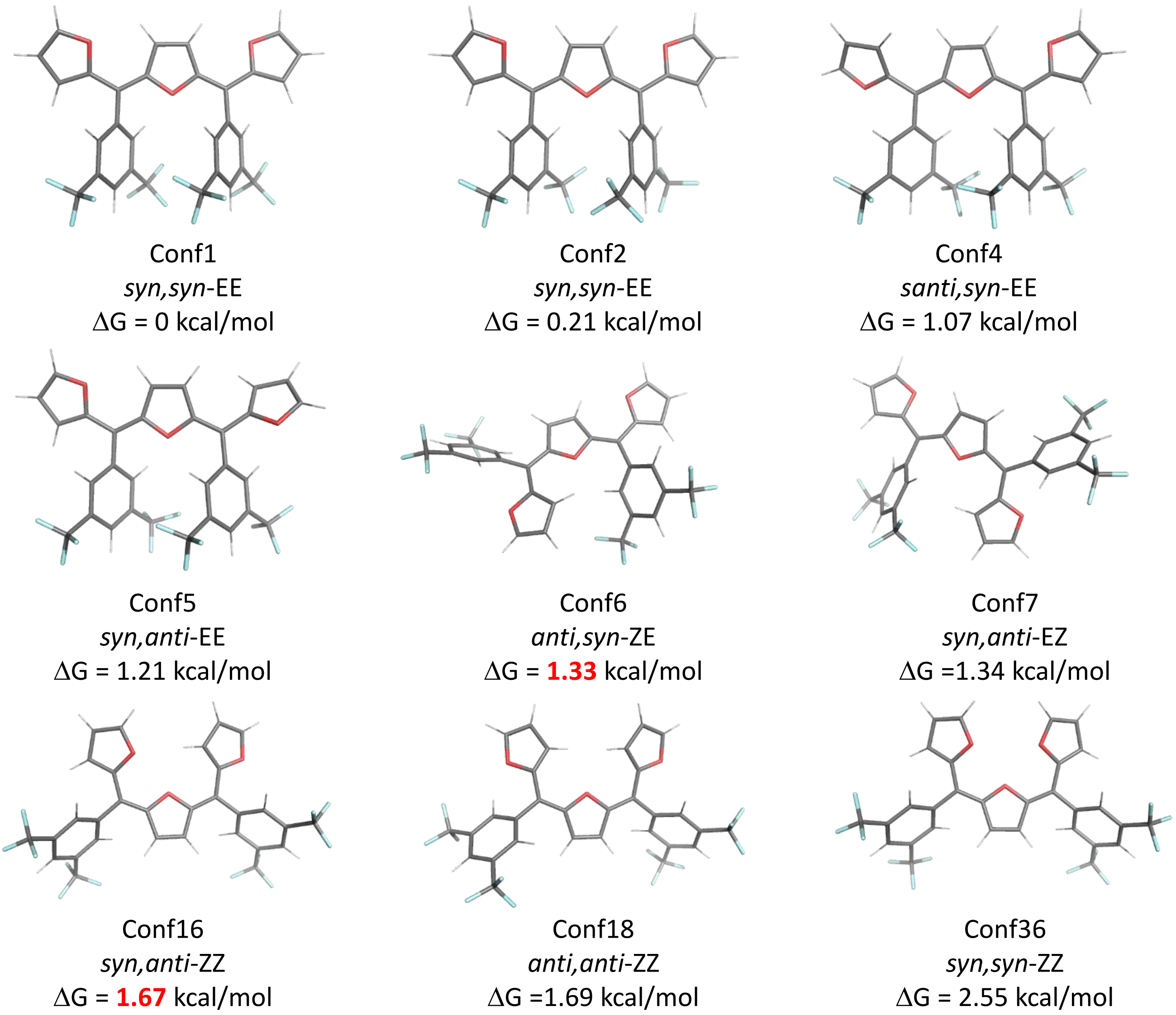

Figure S25. Selected conformers of **1O** obtained by GOAT/CENSO.

Table S4. Summary of the conformation analysis of **1S** by the CENSO/GOAT program.

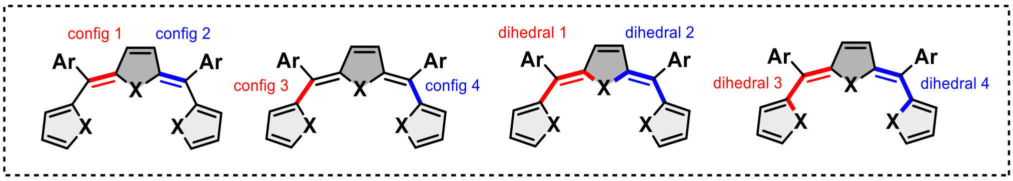

CONF No.	config 1,2	dihedral 1	dihedral 2	config_3,4	dihedral 3	dihedral 4	ΔG_{328K} kcal/mol	P[a]	g[b]	$g{\cdot}P$	proportion (% at 328K)
6	ZZ	3.9	-0.8	syn,syn	17.3	0.1	0	1	1	1	4.788665
4	ZZ	-4.4	4.5	syn,syn	-16.4	16	0.079669	0.884996	1	0.884996	4.237949
2	ZZ	-4.5	4.7	syn,syn	-16.1	15.8	0.176908	0.762396	1	0.762396	3.650861
15	ZZ	6.9	4.5	anti,syn	-149.1	16.9	0.181595	0.756936	2	1.513871	7.249423
3	ZZ	3.2	2.9	syn,syn	17	16.8	0.249975	0.681583	1	0.681583	3.263871
1	ZZ	-2.9	-2.8	syn,syn	-16.9	-16.4	0.27864	0.652271	1	0.652271	3.123508
10	ZZ	-6.4	-4.7	anti,syn	148.2	-17.1	0.284708	0.64623	2	1.292459	6.189155
22	ZZ	6.9	0.2	anti,syn	-150.6	1.6	0.284808	0.64613	2	1.29226	6.188202
5	ZZ	-3	-3.2	syn,syn	-16.7	-16.3	0.304242	0.627158	1	0.627158	3.003251
32	ZZ	8.5	8.2	anti,anti	-148.8	-149.3	0.346304	0.587982	1	0.587982	2.81565
13	ZZ	-6.6	-4.7	anti,syn	148.5	-17.5	0.394396	0.546179	2	1.092358	5.230936
26	ZZ	-7.5	-7.4	anti,anti	149.4	150	0.447302	0.503617	1	0.503617	2.411653
14	ZZ	-3.3	7.6	syn,anti	-13.3	-151.7	0.48728	0.473669	2	0.947338	4.536484
16	ZZ	2.7	-7.7	syn,anti	13.2	150.2	0.492514	0.469883	2	0.939765	4.500222
12	ZZ	3.6	-7.8	syn,anti	14.3	151.4	0.652711	0.367536	2	0.735071	3.52001
30	ZZ	6.7	-7.4	anti,anti	-152.5	151.3	1.029625	0.206195	1	0.206195	0.987398
37	ZZ	6.1	-6.6	anti,anti	-155.8	153.3	1.120927	0.179255	1	0.179255	0.858391
29	ZZ	-7.3	6.1	anti,anti	152.5	-153.9	1.219214	0.154174	1	0.154174	0.73829
17	ZE	-5.1	-165.9	syn,anti	-15.5	-141.7	0.958258	0.230042	2	0.460084	2.203189
18	ZE	3.7	-168	syn,anti	16.6	-139.3	1.029618	0.206197	2	0.412393	1.974814
20	ZE	3.3	-167.8	syn,anti	16.4	-140.1	1.13804	0.174612	2	0.349224	1.672317
42	ZE	6.3	-166.7	anti,anti	-151.6	-138.1	1.181168	0.163437	2	0.326874	1.565291
50	ZE	6.4	-166.4	anti,anti	-151.5	-137.8	1.309024	0.134338	2	0.268677	1.286603
36	ZE	6	-167.8	anti,syn	-149.8	35.3	1.596963	0.086384	2	0.172769	0.827331
11	ZE	-2.4	170.3	syn,syn	-16	-31.5	1.619051	0.083507	2	0.167014	0.799776
39	ZE	7.7	168	anti,syn	-152.6	-34.7	1.72244	0.071264	2	0.142527	0.682515
24	ZE	3.1	-172.1	syn,anti	13.1	-157.5	2.150514	0.036964	2	0.073927	0.354012
68	ZE	9	166.9	anti,syn	-151	-41.8	2.256068	0.031439	2	0.062879	0.301106
63	ZE	-6.1	171.7	anti,anti	150.9	157.4	3.150201	0.00798	2	0.01596	0.076425
21	EZ	-167.4	3.7	anti,syn	-140.7	16.9	0.835969	0.277492	2	0.554985	2.657636
19	EZ	-165.7	-4.6	anti,syn	-142	-14.9	0.851813	0.270831	2	0.541662	2.593839
48	EZ	166.5	-6.1	anti,anti	138.3	151.3	1.144804	0.17281	2	0.34562	1.655059
7	EZ	167.4	5	syn,syn	-32.5	16	1.294936	0.137272	2	0.274544	1.3147
9	EZ	168	5.5	syn,syn	-29.9	14.6	1.299379	0.13634	2	0.27268	1.305774
49	EZ	-166.6	-7.6	anti,anti	-139.8	153.1	1.386621	0.119267	2	0.238534	1.14226
44	EZ	166.9	7	anti,anti	139.2	-154.4	1.404424	0.116055	2	0.23211	1.111498
8	EZ	-168.4	3.9	syn,syn	34.3	16.9	1.485228	0.10253	2	0.205059	0.981959
31	EZ	-167.7	6	syn,anti	34.9	-150.8	1.52003	0.097201	2	0.194402	0.930927
25	EZ	-171.5	3.7	anti,syn	-155.8	12	1.895312	0.054668	2	0.109337	0.523577
33	EZ	168.4	8.2	syn,anti	-33.9	-154	1.909751	0.053471	2	0.106942	0.512111
69	EZ	167.8	-9.9	syn,anti	-33.3	137.2	2.276305	0.030479	2	0.060958	0.291905
23	EZ	170.8	5.2	anti,syn	155.7	16.5	2.284532	0.030097	2	0.060193	0.288246
64	EZ	-171.9	-8.2	anti,anti	-155.8	148.4	2.442087	0.023637	2	0.047274	0.226377
61	EZ	172.3	8.4	anti,anti	156	-148	2.776324	0.014158	2	0.028315	0.135592
52	EE	165.5	-164.8	anti,anti	141	-143.7	1.216365	0.154849	1	0.154849	0.741522
51	EE	-165.3	165.8	anti,anti	-142.1	140.3	1.256495	0.145608	1	0.145608	0.697266
53	EE	-165.6	165.8	anti,anti	-140.4	140.8	1.261854	0.144416	1	0.144416	0.691559
40	EE	166.8	-165.1	syn,anti	-30.3	-140.4	1.665054	0.077819	2	0.155638	0.7453
46	EE	-165	167	anti,syn	-139.8	-32.2	1.781325	0.06511	2	0.130221	0.623584
41	EE	167.3	-163.9	syn,anti	-32.6	-142.3	1.800847	0.06319	2	0.12638	0.605193
55	EE	-167.7	-167.9	anti,anti	-138.5	-138.2	1.979129	0.048075	1	0.048075	0.230213

27	EE	-166.3	165.6	syn,syn	32.4	-29.2	2.268436	0.030849	1	0.030849	0.147725
28	EE	-165.8	166.5	syn,syn	31.3	-33.6	2.399686	0.025225	1	0.025225	0.120793
58	EE	168	-166.6	anti,syn	153.6	32.1	2.708873	0.0157	2	0.031401	0.150368
45	EE	169.4	168	syn,anti	-30.3	137.2	2.731049	0.015175	2	0.030351	0.14534
65	EE	165.7	-170.4	anti,anti	142.8	-156.5	2.799322	0.013667	1	0.013667	0.065447
47	EE	-168.7	-168.2	syn,anti	33.9	-136.2	2.855139	0.012546	2	0.025092	0.120155
59	EE	-170.1	166.5	anti,syn	-156.5	-33	3.28797	0.00646	2	0.01292	0.06187
57	EE	-170.1	167.1	anti,syn	-157.1	-32.5	3.43327	0.00517	2	0.010339	0.049512
54	EE	-168.2	177.7	syn,syn	33.6	-3.2	3.554637	0.004292	1	0.004292	0.020552
35	EE	-169.2	-169.4	syn,syn	35.6	34.2	3.722458	0.003318	1	0.003318	0.015889
34	EE	-169.9	-169.7	syn,syn	32.2	33.5	3.768166	0.003093	1	0.003093	0.014813
67	EE	-173.4	-168.2	anti,anti	-164	-137.2	3.800502	0.002944	1	0.002944	0.014096
38	EE	-169.2	-169.1	syn,syn	35.3	33.8	3.813855	0.002884	1	0.002884	0.013811
60	EE	167.2	172.8	syn,anti	-33.9	159.3	4.407517	0.00116	2	0.002321	0.011114
62	EE	172.1	169.3	anti,syn	158.3	-33.5	4.912556	0.000535	2	0.00107	0.005123

[a] P=exp(-$G \,/\, kT$) [b] degeneracy.

Table S5. Summary of the conformation analysis of **1O** by the CENSO/GOAT program.

CONF_ No	config 1,2	dihedral 1	dihedral 2	config3,4	dihedral 3	dihedral 4	ΔG_{328K} kcal/mol	P[a]	g[b]	$g{\cdot}P$	proportion (% at 328K)
1	EE	175.1	175.2	syn,syn	-14.3	-12.9	0.213291	0.721024	1	0.721024	9.976594
2	EE	-175.1	-174.9	syn,syn	14.5	17.2	0.46063	0.493428	1	0.493428	6.827412
3	EE	175	175.6	syn,syn	-13.6	-12.6	0	1	1	1	13.83669
4	EE	168.2	176.3	anti,syn	148.6	-13.2	1.432775	0.111118	2	0.222235	3.074999
5	EE	176.3	168	syn,anti	-13.5	149.7	1.206175	0.157288	2	0.314577	4.352702
6	EE	168.4	176.2	anti,syn	149.5	-12.1	1.077209	0.191684	2	0.383369	5.304558
7	EE	168.6	176.6	anti,syn	151.1	-14.3	1.438862	0.110085	2	0.22017	3.04643
13	EE	-169.5	-169.4	anti,anti	-150.9	-151.1	2.914483	0.011454	1	0.011454	0.158492
15	EE	-169.5	-169.7	anti,anti	-152.1	-150.9	2.727535	0.015257	1	0.015257	0.211113
16	EE	-169.6	-169.8	anti,anti	-150.1	-151	2.589087	0.018866	1	0.018866	0.261047
17	EE	168.9	-179.3	syn,syn	-16.3	17.4	3.019904	0.009745	1	0.009745	0.134834
18	EE	-179.9	-168.4	syn,syn	-18	17.7	3.137707	0.008134	1	0.008134	0.112549
19	EE	177.9	-169.4	syn,syn	-17.6	15.2	2.691372	0.016127	1	0.016127	0.223151
20	EE	172.2	-176.9	syn,syn	-9.1	18.3	2.461772	0.022934	1	0.022934	0.317329
21	EE	177.8	-171.2	syn,syn	-16.8	10.5	2.636508	0.017543	1	0.017543	0.242738
22	EE	178.8	-169.4	syn,syn	-16	14.6	2.911935	0.011499	1	0.011499	0.159113
34	EE	-162.3	-179.9	anti,syn	-150.2	-17.9	4.033195	0.00206	2	0.004121	0.057014
35	EE	-162.6	179.9	anti,syn	-150.8	-16.3	4.026882	0.00208	2	0.004161	0.057569
39	EE	-171.5	168.9	anti,syn	-141	-15.3	3.090204	0.008749	2	0.017498	0.242108
41	EE	-171.2	168.9	anti,syn	-141.6	-14.7	2.986797	0.010252	2	0.020504	0.283712
42	EE	-172.1	168.5	anti,syn	-137.6	-17.7	3.260592	0.006737	2	0.013474	0.186437
43	EE	170.6	-169.7	anti,syn	142.8	13.2	2.880886	0.01206	2	0.02412	0.333744
56	EE	-172.3	162.6	anti,anti	-139.2	151.6	4.077328	0.001925	1	0.001925	0.026642
10	EZ	-172.7	-4.2	syn,anti	17.2	169.1	1.337632	0.128572	2	0.257144	3.558029
11	EZ	-172.1	-3.8	syn,anti	17.7	172.2	1.383057	0.119921	2	0.239841	3.318612
12	EZ	-172.7	-4	syn,anti	17.2	170.2	1.346511	0.126833	2	0.253667	3.50991
23	EZ	-169.1	3.9	anti,anti	-148.3	-164.8	2.134256	0.037897	2	0.075793	1.04873
24	EZ	-168.9	4.6	anti,anti	-149.1	-162.8	2.296078	0.029568	2	0.059137	0.81826
28	EZ	169.2	-4.3	anti,anti	148.7	164.1	2.062042	0.042335	2	0.084669	1.171542
31	EZ	-176.3	4.4	syn,syn	16.8	15.3	1.605491	0.085262	2	0.170524	2.359486
33	EZ	172	2.6	syn,syn	-17.1	9.3	1.710272	0.072606	2	0.145212	2.009249
50	EZ	169.6	-3.7	anti,syn	148.6	-16	2.496454	0.021746	2	0.043492	0.601786
8	ZE	4.2	-176	anti,syn	-164.8	17.2	1.427993	0.111935	2	0.223871	3.097629
9	ZE	4.8	-175.7	anti,syn	-163.1	17.1	1.332097	0.129668	2	0.259336	3.588356
14	ZE	-3.5	-172.3	anti,syn	173	17.2	1.466635	0.105495	2	0.21099	2.919404
25	ZE	4.2	-176.3	syn,syn	15.7	16.7	1.819472	0.061411	2	0.122822	1.699448
26	ZE	4.6	166.3	anti,anti	-167.4	150.2	1.963761	0.049221	2	0.098442	1.362109
27	ZE	-5.5	-172.5	syn,syn	-12.8	19.4	1.395733	0.117612	2	0.235224	3.254727
30	ZE	-3.7	-166.3	anti,anti	170.8	-149.8	2.137876	0.037687	2	0.075374	1.042924
48	ZE	-4.9	-166.7	syn,anti	-13.4	-147.4	1.95598	0.049812	2	0.099623	1.37846
49	ZE	-3.2	168.9	syn,anti	-13.7	147.1	2.175213	0.03559	2	0.071179	0.984887
51	ZE	4	-169.8	syn,anti	16.6	-148.9	2.588209	0.018892	2	0.037783	0.522799
55	ZE	-2.6	-165.8	syn,anti	-10.8	-150.3	2.618116	0.018045	2	0.03609	0.499363
29	ZZ	7	6.8	anti,anti	-163.6	-163.8	1.94744	0.050468	1	0.050468	0.698316
32	ZZ	7	6.9	anti,anti	-163.3	-163.3	1.842181	0.059309	1	0.059309	0.820641
36	ZZ	-0.5	10.6	anti,anti	175.2	-155.8	2.447891	0.023427	1	0.023427	0.324157
37	ZZ	10.6	-0.8	anti,anti	-156.6	174.9	2.28664	0.03	1	0.03	0.415094
38	ZZ	-5.8	6.3	syn,anti	-15.9	-167	1.667865	0.077484	2	0.154969	2.144255
40	ZZ	-4.5	-4.3	syn,anti	-13.9	166.7	1.673412	0.076828	2	0.153656	2.126092
46	ZZ	-5.7	6.5	syn,anti	-15.9	-166.5	1.714671	0.072118	2	0.144235	1.995741
47	ZZ	-3.8	-4.8	anti,syn	164.9	-16.4	1.905786	0.053797	2	0.107595	1.488755
52	ZZ	3.6	4	anti,anti	-174.4	-173.8	4.288548	0.001393	1	0.001393	0.01927
53	ZZ	4.7	5.5	anti,anti	-165.8	-164.5	1.687732	0.075159	1	0.075159	1.039957
54	ZZ	4.1	3.8	anti,anti	-174.1	-174	4.409688	0.001157	1	0.001157	0.016003
58	ZZ	4.2	4	syn,syn	16.4	15.8	2.752943	0.014674	1	0.014674	0.203045
59	ZZ	-6.5	6.4	syn,syn	-15.9	15.5	2.591277	0.018803	1	0.018803	0.260172
60	ZZ	-7.2	6.9	syn,syn	-17.2	16.8	2.553213	0.019933	1	0.019933	0.275811

[a] P=exp(-G ⁄ kT) [b] degeneracy.

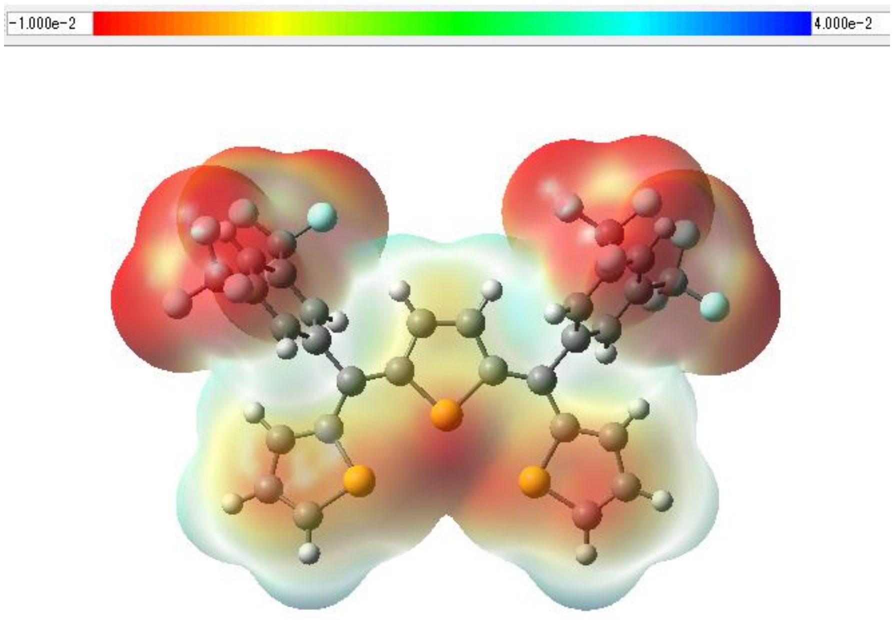

Figure S26. Electrostatic potential surface of *syn,syn*-*ZZ*-**1S**.

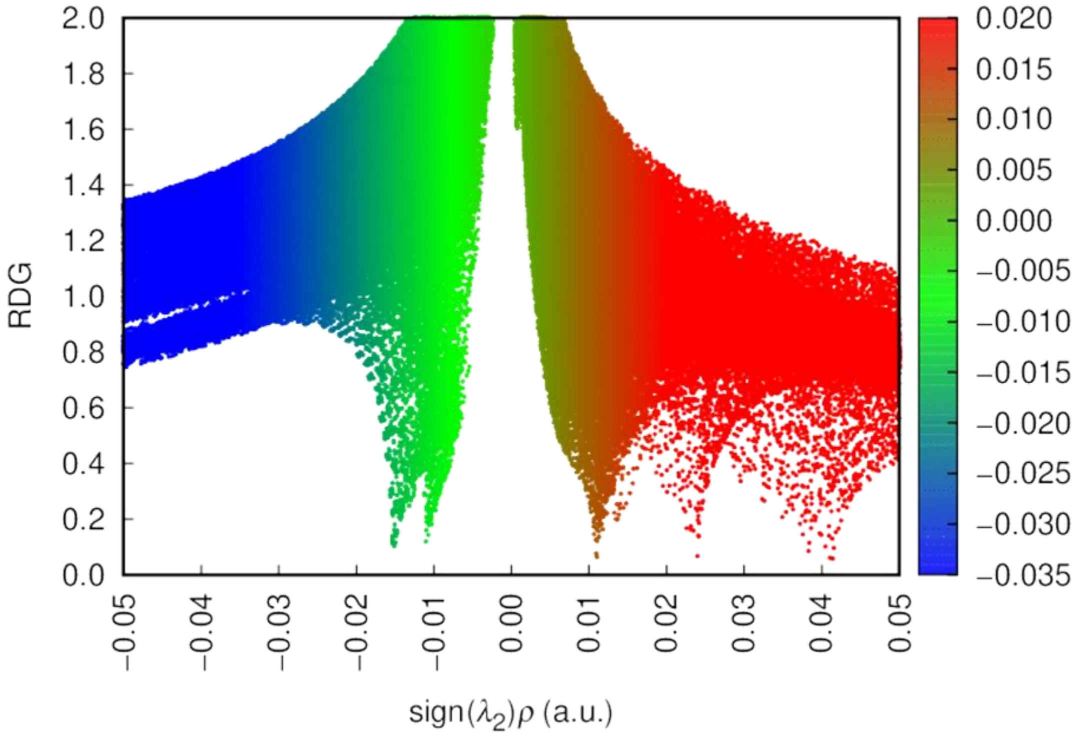

Figure S27. NCI RGB scatter plot of *syn,syn*-*ZZ*-**1S**.

Table S6. Cartesian coordinates for the optimized structure of *syn,syn*-*ZZ*-**1S**.

Stoichiometry: $C_{30}H_{14}F_{12}S_3$, full point group: Cs, charge: 0, multiplicity: 1.
Total energy: −3545.51696047 a.u., No. of imaginary freq: 0.

Center No.	Atomic No.	Coordinates (Angstroms)		
		X	Y	Z
1	6	0.936922	-0.189537	1.281494
2	6	0.936922	-0.189537	-1.281494
3	6	1.202863	-0.252367	2.622047
4	6	1.202863	-0.252367	-2.622047
5	6	2.478871	-0.517939	3.244051
6	6	2.682231	-0.822992	4.580234
7	6	4.03684	-1.020345	4.919935
8	1	1.871644	-0.910894	5.286634
9	6	4.874475	-0.867633	3.850647
10	1	4.378309	-1.268619	5.914095
11	6	2.478871	-0.517939	-3.244051
12	6	2.682231	-0.822992	-4.580234
13	6	4.03684	-1.020345	-4.919935
14	1	1.871644	-0.910894	-5.286634
15	6	4.874475	-0.867633	-3.850647
16	1	4.378309	-1.268619	-5.914095
17	6	0.069999	-0.033545	-3.564737
18	6	-1.00031	-0.920251	-3.611541
19	6	0.070256	1.065842	-4.425512
20	6	-2.058452	-0.705314	-4.490438
21	1	-1.003359	-1.784948	-2.961903
22	6	-0.985386	1.273933	-5.299608
23	1	0.902434	1.755052	-4.406731
24	6	-2.060618	0.390709	-5.337387
25	1	-2.878822	0.552729	-6.022417
26	6	0.069999	-0.033545	3.564737
27	6	0.070256	1.065842	4.425512
28	6	-1.00031	-0.920251	3.611541
29	6	-0.985386	1.273933	5.299608
30	1	0.902434	1.755052	4.406731
31	6	-2.058452	-0.705314	4.490438
32	1	-1.003359	-1.784948	2.961903
33	6	-2.060618	0.390709	5.337387
34	1	-2.878822	0.552729	6.022417
35	6	-3.195705	-1.690952	-4.497665
36	6	-0.998707	2.494771	-6.180265
37	9	-2.765536	-2.941331	-4.770173
38	9	-3.804804	-1.746527	-3.292748
39	9	-4.137233	-1.385283	-5.406416
40	9	-1.6257	3.530611	-5.578853
41	9	0.244551	2.913284	-6.483391
42	9	-1.642583	2.270786	-7.34198
43	6	-3.195705	-1.690952	4.497665
44	6	-0.998707	2.494771	6.180265
45	9	-2.765536	-2.941331	4.770173
46	9	-4.137233	-1.385283	5.406416
47	9	-3.804804	-1.746527	3.292748
48	9	0.244551	2.913284	6.483391
49	9	-1.642583	2.270786	7.34198
50	9	-1.6257	3.530611	5.578853
51	6	-0.319833	0.169366	0.675059
52	1	-1.179374	0.433767	1.270739
53	6	-0.319833	0.169366	-0.675059
54	1	-1.179374	0.433767	-1.270739
55	1	5.947565	-0.962604	3.824255
56	1	5.947565	-0.962604	-3.824255
57	16	4.017198	-0.4799	2.417464
58	16	2.108439	-0.519052	0
59	16	4.017198	-0.4799	-2.417464
